# Increasing benefits in one-time public goods does not promote cooperation

**DOI:** 10.1073/pnas.2410326121

**Published:** 2024-10-04

**Authors:** Natalie Struwe, Esther Blanco, James M. Walker

**Affiliations:** ^a^Department of Public Finance, University of Innsbruck, Innsbruck 6020, Austria; ^b^The Ostrom Workshop, Indiana University, Bloomington, IN 47408; ^c^Department of Economics, Indiana University, Bloomington, IN 47405

**Keywords:** cooperation, public goods, marginal per capita return, social dilemma, experiments

## Abstract

The long tradition of research on cooperation includes a well-established finding that individuals respond to the degree of conflict between self- and collective interests (that is, the relative benefits from cooperation) in providing public goods. Existing empirical evidence builds upon settings where participants make multiple decisions or strategically consider alternative scenarios. Here, we consider a decision setting where participants face a one-time (single-decision) setting. One-time cooperative encounters often occur in volunteering or donating to immediate needs for crisis relief. For these distinct and highly relevant settings, we report a lack of responsiveness to increases in cooperation benefits, thereby highlighting limits to our understanding of the determinants of one-time cooperation encounters. Across two studies, n = 2,232 individuals participate in treatments where we vary across participants the relative benefit from contributing to a public good (that is, the marginal per capita return, the MPCR). We examine decisions from alternative participant pools (UK general population vs. students), implementations varying the physical distance between participants (online vs. in the laboratory), and more complex decision settings considering group-to-group interactions including not only providers but also donors to public goods. Throughout, neither average contribution levels, nor the distribution of contributions are significantly affected by the increases in cooperation benefits. The mechanism behind these results can be explained by the close correlation between expectations of other’s cooperation and own cooperation, and the fact that these expectations do not increase with higher benefits from cooperation.

## Significance

Cooperation is a key feature for well-functioning societies. The fundamentals of cooperative behavior have been widely studied, with an emphasis on repeated interactions within groups. One-time cooperative interactions are fundamentally different, pervasive, and highly relevant, for example, when people make decisions on volunteering or donating to immediate needs for crisis relief. We provide evidence for a lack of responsiveness to large increases in the benefits from one-time (single-decision) cooperation. This surprising result illustrates limits to our understanding of the fundamentals of cooperative behavior in one-time provision of relevant, and social welfare enhancing, public goods and provides strong evidence for the need for further research into cooperation in such stark settings.

The provision of public goods often relies on individuals to leverage collective provision efforts while facing one-time encounters. Such settings may include decisions to contribute to immediate public emergencies, including disaster relief efforts such as donating money, blood, food, or shelter during natural disasters, war, or emergencies; or volunteering activities in clean-up events such as oil spills, tree-planting initiatives after wild-fires, or help in processing paperwork for war refugees, or other forms of active hands-on hours of work. A prominent example for this type of collective action was the widespread displayed prosocial response to the aftermath of Hurricane Katrina in 2005 (1) that included many people traveling to affected regions to help others in need. These empirical observations are consistent with results from highly controlled decision environments in the experimental laboratory. Even in situations where individuals only interact with anonymous group members a single time, a substantial share of participants choose to make positive, costly, contributions to public goods (see among others, 2–6). This evidence points to cooperation being a fundamental component of human behavior, emerging in the field and laboratory settings.

Studying true one-time (single) decision environments therefore is a highly relevant endeavor, both for the real-world analogues and because the one-shot game can be viewed as a fundamental building block for more complex settings. One-time cooperation settings have distinct features: Individuals often have little to no information on other’s preferences, less prevalent reputational concerns, and no long-term relationships. Previous evidence suggests that in such settings individuals tend to rely strongly on their own personal normative judgments (7) as well as on altruistic motivations and beliefs about others’ behavior (5) to make contribution decisions to public goods. Yet, many open questions remain for these relevant one-time public good settings, including the determinants of the degree of cooperation. In this paper, we examine whether the willingness to cooperate in the provision of public goods increases when relaxing the tension between self- and social interests in one-time (single) cooperation decisions. We report on two experimental studies designed to test whether cooperation increases in the relative benefits from one-time public good provision.

The long history of experimental research studying the determinants of cooperation in public good environments has provided strong evidence, for various decision settings, that cooperation levels increase in the benefits from public goods (the so-called Marginal Per Capita Return—MPCR—the marginal benefit from a public good relative to the marginal cost of providing it). This metric provides a measure of the tension between self-interest and collective interest in a public good social dilemma. The positive relationship between benefits from repeated public goods and public good provision levels was first established by Isaac et al. (8) studying decisions in a setting they referred to as the Voluntary Contribution Mechanism (herein VCM). Their results have been replicated numerous times [(9–22); for reviews, see also 23–25]. The responsiveness of cooperation to the benefits from cooperation has been shown to be robust to numerous alternative experimental designs to elicit cooperative behavior, including within- and between-subject changes in the MPCR, strategy method and direct responses, partner and stranger matching protocols, small and large groups, homogeneous and heterogenous MPCRs within groups, provision and appropriation frames (26–28). We provide an extended discussion of related literature in *SI Appendix*, section 1. The previous studies have in common that they either address behavior in repeated decision environments or in environments where individuals are asked to strategically consider alternative scenarios. In the former, individuals have the opportunity to be forward-looking, learn about other group members’ behavior, and build trust relationships based on reciprocal behavior over time. In the latter, individuals make contribution decisions for various possible levels of the MPCR. Recently, two related studies show significant cooperation responses to changes in the MPCR with random rematching within groups across multiple decision rounds (22) or a single contribution decision following a first stage where participants make decisions based on hypothetical contributions of other group members (29). These studies deviate from the pure one-time (single) decision settings we consider here, in so far as repetition of decisions in the former study (even with rematching within groups) may influence participants’ strategic thinking beginning in the firstperiod due to norms of reciprocity and forward-looking behavior, and in the latter study, first strategically considering ones’ conditional responses to all possible levels of others’ cooperation may subsequently influence participants’ unconditional contribution decision.

In addition, binary choice Prisoner Dilemma (PD) games provide further evidence on studying cooperation in social dilemma games to that of the VCM. Pertinent to our study, two studies report results for decision-making in PD games in a one-time (single) decision setting, varying the benefits from mutual cooperation (30, 31). They find a strong positive relationship between changes in game parameters for mutual cooperation and cooperation levels, as well as with expectations of other’s choices. The difference in results between these studies and ours adds to the literature on differences in behavior between these two game settings. In particular, the prior literature includes findings of differences in cooperation rates between VCM and PD games due to the limited choice set in the PD vs. VCM setting (32), differences in the framing of these games (33, 34), differences in responses to changes in group size (33) and to finitely vs. indefinitely repeated games (17). These results provide evidence that the VCM one-time (single) setting deserves separate investigation as one cannot simply extrapolate from PD games to VCM settings.

We present results on one-time (single) cooperation responses to variations in the tension between self-interest and collective interest (changes in the MPCR) in a VCM setting where participants have little to no information about other people’s preferences and where reputational concerns and long-term relationships do not exist. We further report results on mediating effects through the expectations of others’ contributions and self-reported motivations for cooperation. We present results for two preregistered studies including a total of 2,232 participants in eight between-subjects treatment conditions varying the benefits from cooperation. Study 1 was motivated by the (unexpected) results from Study 2, showing a lack of responses to changes in the MPCR. We consider Study 1 as the primary study due to its simplicity and closeness to the broad literature on responses to the benefits from cooperation in VCM games for other decision settings (e.g., repeated decisions, strategy method, etc.). Study 1 (n = 952) examines a standard VCM where participants make a single contribution decision (direct responses) out of their initial endowment, with no interaction with other group members. Participants also provide an expectation of the average contribution of others in their group (prior to contributions in the primary experiments and after in robustness experiments).

**Fig. 1.**
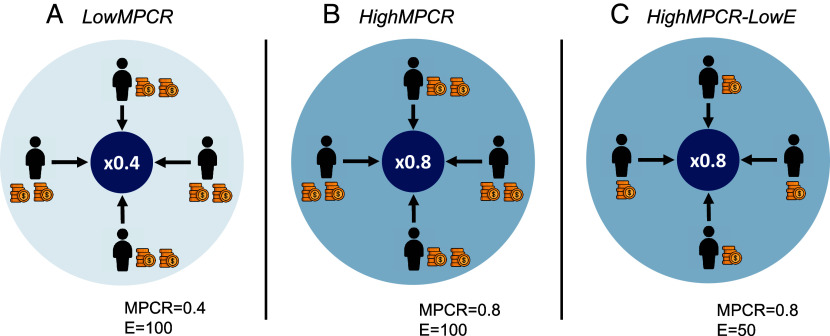
Experimental design study 1. Panel *A*: *LowMPCR* treatment, with endowment of 100 ECUs and MPCR of 0.4. Panel *B*: *HighMPCR* treatment with endowment of 100 ECUs and MPCR of 0.8. Panel *C*: *HighMPCR-LowE* treatment, with endowment of 50 ECUs and MPCR of 0.8. In all treatments participants face a single-decision linear public good environment in groups of four. Each member receives an endowment (E) in ECUs (Experimental Currency Units) that can be used to make continuous contributions (in increments of 1 ECU) to a Group Account. The Group Account constitutes a public good with an equal marginal return (MPCR) for all group members. The shading indicates the marginal benefit to all members of a group from a unit contribution to the public good, with stronger shading highlighting the larger collective benefit with a higher MPCR = 0.8 and the lighter shading the lower MPCR = 0.4.

Across three treatment conditions (Fig. 1), we vary the benefits from cooperation (MPCR) in the provision of the public good: (Panel *A*) a low value of MPCR = 0.4 [*LowMPCR*], (Panel *B*) a high value of MPCR = 0.8, holding constant the individual endowment [*HighMPCR],* and (Panel *C*) a high value of MPCR = 0.8 halving the individual endowment [*HighMPCR-LowE]*. Note that relative to *LowMPCR*, the maximum gains from full cooperation are doubled in *HighMPCR*, but payoffs at the “selfish” equilibrium of zero contributions are constant. In contrast, relative to *LowMPCR*, the maximum gains from full cooperation are held constant in *HighMPCR-LowE,* but payoffs at zero contributions are halved. The latter two treatments follow the approach of Isaac et al. (8) and Isaac and Walker (9), as increasing the MPCR without changing the endowment increases maximum gains from cooperation. Including both treatments in this study provides a complete and thorough test of the responses to changes in the benefits from cooperation. Study 1 includes three alternative data collection processes (herein, *samples*), including a sample from the UK general population from an online experiment using Prolific (*general population*, n = 232), an online experiment with university students in Austria (*students [online],* n = 240), and a conventional laboratory experiment with university students in Austria (*students [lab]*, n = 244). We also include a robustness condition for the UK general population (*rob*, n = 236) changing the order of contributions and expectations.

**Fig. 2.**
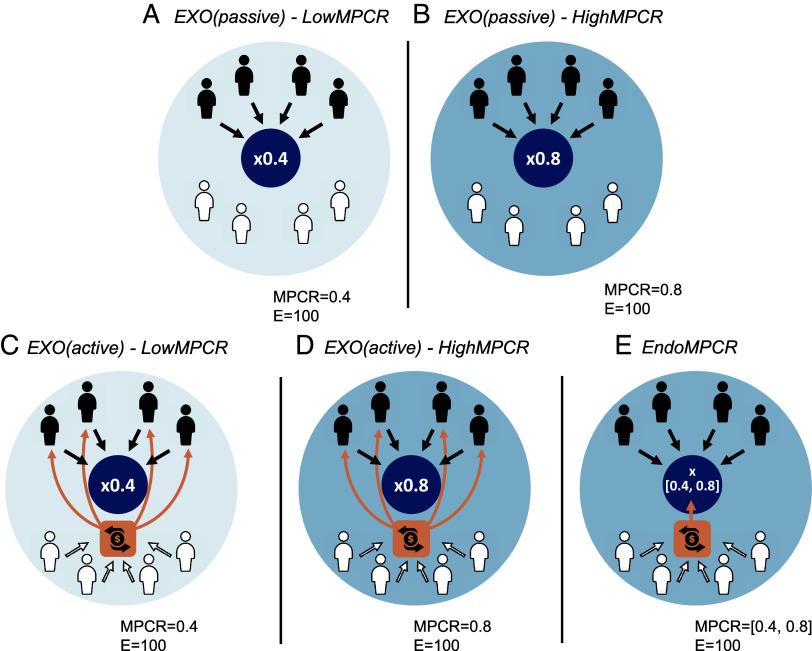
Experimental design study 2. Panel *A*: *EXO(passive)-LowMPCR* treatment with MPCR of 0.4 and passive beneficiaries. Panel *B*: *EXO(passive)-HighMPCR* treatment with MPCR of 0.8 and passive beneficiaries. Panel *C*: *EXO(active)-LowMPCR* treatment with MPCR of 0.4 and beneficiaries make cash donations to providers. Panel *D*: *EXO(active)-HighMPCR* treatment with MPCR of 0.8 and beneficiaries make cash donations to providers. Panel *E*: *EndoMPCR* treatment with MPCR being endogenously defined by beneficiaries’ transfers in the interval of 0.4 to 0.8. In all treatments, participants face a single-decision linear public good environment in groups of eight members, four providers (black) and four beneficiaries (white). Providers receive an endowment (E) of 100 ECUs that can be used to make continuous contributions (in increments of 1 ECU) to a Group Account. The Group Account constitutes a public good with an equal marginal return (MPCR) for all group members. The shading indicates the marginal benefit to all members of a group from a unit contribution to the public good, with stronger shading highlighting the larger collective benefit with a higher MPCR = 0.8 and the lighter shading the lower MPCR = 0.4. Beneficiaries cannot make contributions to the public good but benefit from public good provision. In all treatments with active beneficiaries, beneficiaries can use their endowment to make a donation to a Transfer Account. In *EXO(active)* treatments, the Transfer Account is shared equally among providers. In the *EndoMPCR* treatment, the Transfer Account is used to increase the MPCR from public good provision.

Study 2 (n = 1,280) considers a more complex decision setting, including not only providers of the public good (“providers,” n = 640, our focus) but also additional beneficiaries (“beneficiaries,” n = 640) who in some treatment conditions can make donations to providers. Both providers and beneficiaries benefit from public good provision, but beneficiaries cannot contribute to the provision of the public good. The decision setting for this study aims to capture the reality that many public goods are provided by a subgroup of society that undertake costly actions which benefit a broader segment of society who can (in some cases) make donations to support public good provision by others. These donations often take the form of direct cash-transfers but can also be used to increase the marginal benefit of efforts by public good providers (e.g., in-kind donations such as protective equipment, tools, or gadgets). In Study 2, we aim to understand how changes in the marginal benefits from public goods impact cooperativeness in the presence of not only providers but also potential donors. Study 2 includes five treatment conditions (Fig. 2). Across four treatments, we exogenously vary the MPCR from MPCR = 0.4 to MPCR = 0.8, equivalent to Study 1. In two of those treatments, beneficiaries are passive and cannot make donations [*EXO(passive)-LowMPCR* and *EXO(passive)-HighMPCR*] (Panels *A* and *B*). In two additional treatments beneficiaries can make donations in the form of a cash transfer to providers [*EXO(active)-LowMPCR* and *EXO(active)-HighMPCR*] (Panels *C* and *D*). We also examine an additional treatment where donations from beneficiaries endogenously increase the MPCR [*EndoMPCR*] (Panel *E*). Study 2 considers a sample from the UK general population from an online experiment using Prolific.

In all treatments in Study 1 and Study 2, the marginal benefit of the public good is smaller than one, such that the individual return from a unit contribution is lower than the cost of that contribution. In addition, the cumulative value of a unit contribution (that is, the marginal benefit to all group members) exceeds the cost of that contribution. Thus, all decision settings constitute a social dilemma, in which there exist conflicts of interest (weakened for higher MPCRs) between self- and social welfare concerns, resulting in free-riding incentives.

In both Study 1 and Study 2, the (sub-)sample sizes are calibrated to detect meaningful effect sizes of 15%-points differences in individual contributions to the public good [based on an a priori power analysis for two-sample means tests, conventional significance α = 0.05 and power β = 0.8 levels, using data from a previous public good experiment in (35) that are well below the typical range of effect sizes for comparable large increases in the MPCR previously reported in the literature; first-period effect sizes ranging between 17 to 30%-points (see references and details in *Materials & Methods* section). Pooling the data in Study 1, our sample size enables us to exclude small effects of 8%-points, and for study 2, we can exclude small effects of 11 to 13%-points, dependent on the specific comparison. These minimum detectable effect sizes are estimated based on our collected data, for two-sample means tests (as preregistered) with conventional significance α = 0.05 and power β = 0.8 levels.

In the following, we report results on treatment effects based on average contributions (pre-registered, relying on OLS (Ordinary Least Squares) regressions; see *SI Appendix*, section 3.2 for tests of OLS model assumptions). For Study 1 we examine effects both pooling across all samples and within samples, similarly for average expectations (exploratory, relying on OLS regressions), and the distributions of contributions and expectations (exploratory, relying on Kolmogorov–Smirnov tests for equality of distributions). Similarly, for Study 2, we examine effects on average cooperation comparing *EXO(passive)* and *EXO(active)* treatments (pre-registered, OLS regressions), comparisons of groups in *EndoMPCR* (exploratory, OLS regressions), the effect on average expectations (exploratory, OLS regressions), and distributions of contributions and expectations (exploratory, Kolmogorov– Smirnov tests).

## Results—Study 1

### Increasing Benefits from Cooperation in the One-Time (Single) Decision Public Goods Game Does Not Affect Cooperation Levels Nor Expectation Levels.

**Fig. 3.**
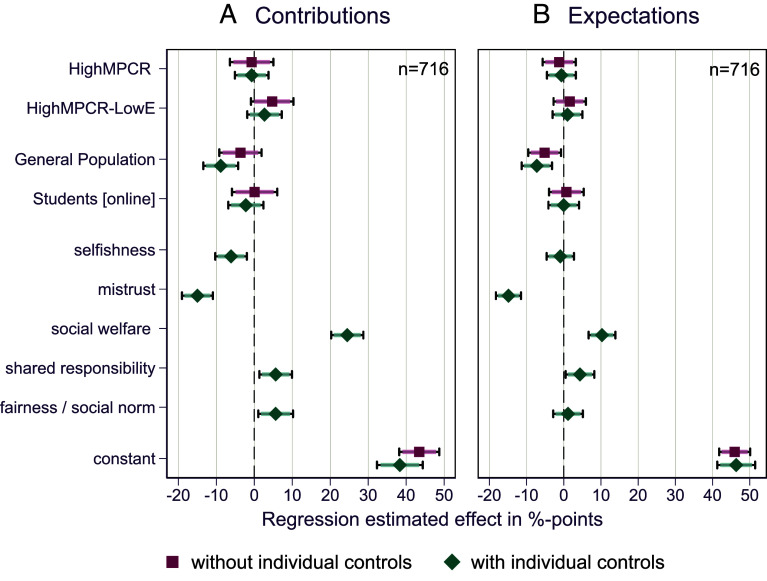
Point estimates and CI (90% CI indicated by shaded spikes, and 95% CI indicated by the “caps” at the ends of each side of the CI) from OLS regressions with robust SE for the main behavioral outcomes in Study 1: contribution to the public good in percentage of endowment (*A*) and individual expectations of the behavior of others in percentage of endowment (*B*). Explanatory variables in models without individual controls are the treatment dummies (*HighMPCR and HighMPCR-LowE)* indicating the effect of the respective treatment condition as compared to the *LowMPCR* condition; and the population dummies (*General Population* and *Students [online]*) indicating the effect of the population sample as compared to *Students [lab]*. In models with individual controls, we include self-reported motivations from postexperimental questionnaires, included as dummy variables that take the value 1 if the participant indicated the respective motivation, and 0 otherwise. Constant indicates the mean of the respective dependent variable for the *LowMPCR* condition. See *SI Appendix*, Tables S3 and S4 for the full regression outputs behind this figure.

On average and pooling across samples, we find no significant difference in contribution levels when increasing the MPCR = 0.4 (*LowMPCR*) to MPCR = 0.8 (*HighMPCR* or *HighMPCR-LowE*), at conventional *P*-values > 0.05 (Fig. 3*A*). Without individual controls from questionnaire data, average contributions to the public good in *HighMPCR* are 0.69%-points insignificantly *lower* than in *LowMPCR* (Fig. 3*A*: *P*-value = 0.813; 95%-CI: −6.42, 5.04), while they are 4.72%-points insignificantly higher in *HighMPCR-LowE* than in *LowMPCR* (Fig. 3*A*: *P*-value = 0.098; 95% CI: −0.88, 10.33). Once adding motivations as individual controls, the effect size remains virtually unchanged for *HighMPCR* compared to *LowMPCR* (Fig. 3*A*: ß = −0.69; *P*-value = 0.76; 95% CI = −5.11, 3.73), while it is reduced for *HighMPCR-LowE* compared to *LowMPCR* to 2.68%-points and still insignificant (Fig. 3*A*: *P*-value = 0.24; 95% CI: −1.86, 7.23). Finally, the average treatment effect in *HighMPCR* does not significantly differ from that in *HighMPCR-LowE* for *P*-values < 0.05 (*F* = 3.25, *P*-value = 0.07 from the postestimation Wald test without individual controls, and *F* = 1.44, *P*-value = 0.23 with individual controls).

Thus, our results provide strong evidence for a lack of a responsiveness in cooperation to large changes of the benefits from cooperation (*doubling* MPCR from 0.4 to 0.8) in a single-decision public good environment. Based on a sample size of over 700 independent observations in these comparisons, we can exclude small effect sizes of 8%-percentage points. In addition, as compared to the *LowMPCR* condition, we report insignificant *decreases* in cooperation for MPCR = 0.8 when holding endowment constant and insignificant *increases* for MPCR = 0.8 adjusting the endowment to hold maximum efficiency constant. Therefore, the effect is not only statistically undetectable given our sample size but does not have a stable sign in the expected direction of increasing cooperation when increasing the benefits from cooperation.

**Fig. 4.**
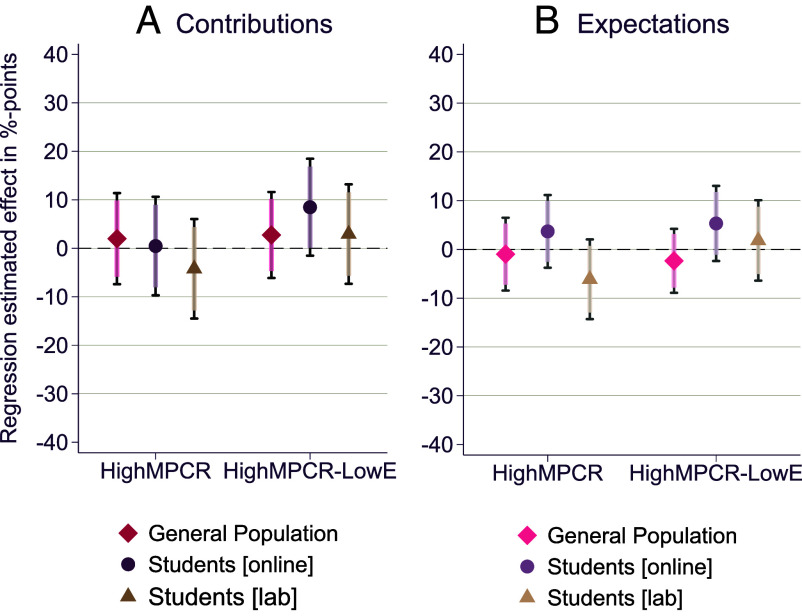
Point estimates and CI (90% CI indicated by shaded spikes, and 95% CI indicated by the caps at the ends of each side of the CI) from OLS regressions with robust SE. Panel *A*: Average treatment effects on individual contributions relative to the *LowMPCR* condition, estimated in a separate model for each sample. The dependent variable in each model is an individual’s contribution to the public good in percentage of endowment and the explanatory variable is a dummy variable indicating the treatment condition. Panel *B*: Average treatment effects on individual expectations of the behavior of others relative to the *LowMPCR* condition, estimated in a separate model for each sample. The dependent variable in each model is an individual’s expectations regarding the average contribution of the other group members, in percentage of endowment, and the explanatory variable is a dummy variable indicating the treatment condition. n = 232 for the General population sample, n = 240 for the students [online] sample, and n = 244 for the *students [lab]* sample. See *SI Appendix*, Tables S5 and S6 for the full regression outputs behind this figure.

These overall results also hold true for each sample separately, as shown in Fig. 4 comparing *LowMPCR* to *HighMPCR* and to *HighMPCR-LowE* (*general population* sample *P*-values: 0.68 and 0.54 respectively; *students [online]* sample *P*-values: 0.93 and 0.096 respectively; *students [lab]* sample *P*-values: 0.42 and 0.57 respectively. See *SI Appendix*, Tables S5 and S6 for further details).

It is worth emphasizing that the lack of treatment effects is not associated with untypically low cooperation levels. Average contributions to the public good range between 40 to 50% of endowment across treatment conditions and samples (see *SI Appendix*, Table S1 for overview statistics of average individual contributions and SD). These contribution levels are within the typical range of values previously reported in the literature for an MPCR value of 0.4 in groups of four, and well above the zero-level predicted for groups composed of individuals with purely selfish preferences. We further observe that average contributions in the general population sample generally do not differ significantly from those of the conventional student sample, in the laboratory (Fig. 3*A* without individual controls: ß = 0.08; *P*-value = 0.98; 95% CI: −5.88, 6.05) or online (Fig. 3*A* without individual controls: ß = −3.65; *P*-value = 0.20; 95% CI: −9.22, 1.92).

When exploring the mechanisms for the lack of responsiveness to the changes in the benefits from cooperation, we find that on average, there is no significant difference in expectations about other’s cooperation when increasing the MPCR from 0.4 to 0.8 (in both treatments), at conventional *P*-values > 0.05 (Fig. 3*B* and *SI Appendix*, Tables S2 and S4. *HighMPCR* without individual controls: ß = −1.24; *P*-value = 0.59; 95% CI: −5.69, 3.22; *HighMPCR-LowE* without individual controls: ß = 1.60; *P*-value = 0.47; 95% CI: −2.75, 5.95). The beliefs of others’ contributions were on average largely accurate, with estimates of 39 to 49% of endowment (*SI Appendix*, Table S2).

It is remarkable that there are significant correlations between self-reported motivations for cooperation (from a postexperimental questionnaire) and observed behavior, in line with the systematic behavior that we would expect if participants understood the game the way social scientists do: both selfish motivations as well as mistrust are negatively correlated with contributions (Fig. 3*A*: ß = −6.12; *P*-value = 0.004; 95% CI: −10.28, −1.96 for selfishness, and ß = −14.98; *P*-value < 0.0001; 95% CI: −19.05, −10.90 for mistrust), while there is a positive association with being motivated by increasing social welfare (Fig. 3*A*: ß = 24.49; *P*-value < 0.0001; 95% CI: 20.25, 28.72), feelings of shared responsibility (Fig. 3*A*: ß = 5.63; *P*-value = 0.01; 95% CI: 1.34, 9.93) and considerations of fairness and social norms to contribute (Fig. 3*A*: ß = 5.63; *P*-value = 0.02; 95% CI: 1.06, 10.20). Importantly, though, we find no significant differences in how these motivations affect behavior across MPCR levels (that is, no significant interaction effects between individual motivations and treatments; see *SI Appendix*, Table S3). Further, across treatments and samples, there is no systematic difference in the frequency for these self-reported motivations (*SI Appendix*, Figs. S1 and S2).

Finally, we address whether the order of decision-making between eliciting expectations and contributions is driving the observed nonresponsiveness to changes in the public good benefit. We find that the order of eliciting expectations does not systematically affect results (see detailed description of robustness treatments in *SI Appendix*, section 2, and full set of analysis in *SI Appendix*, section 5, Figs. S8 and S9, and Tables S13–S16). See also *SI Appendix*, section 6 where we report *P*-values for treatment comparisons after correcting for multiple hypothesis testing (*SI Appendix*, Table S17).

### Variations in Benefits from One-Time Cooperation Do Not Affect the Distributions of Either Contributions or Expectations.

**Fig. 5.**
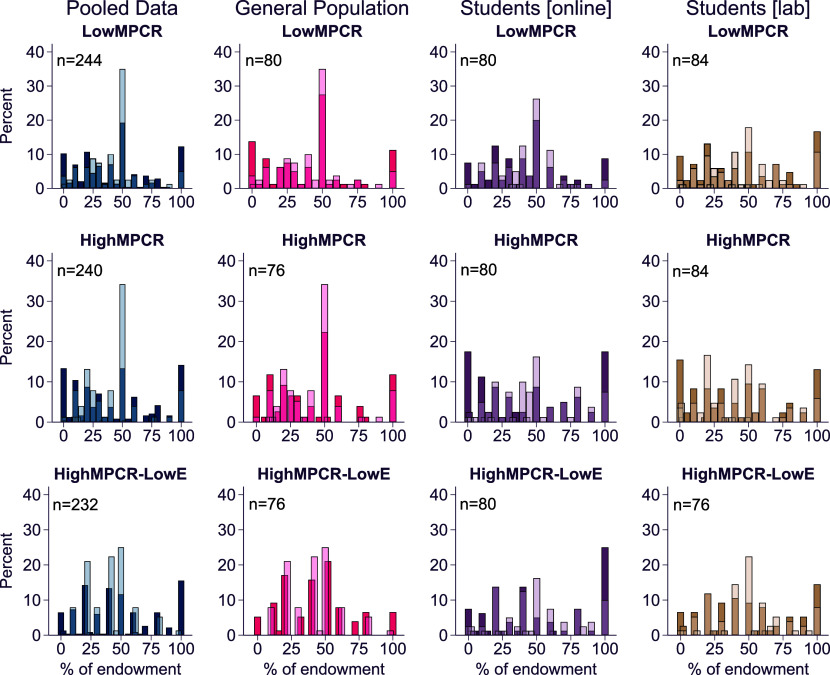
Distributions of individual contributions in percent of endowment (dark-shaded) and individual expectations of behavior of others in percent of endowment (light-shaded), for each treatment and each sample separately, as well as for the pooled data.

A high prevalence of unconditional contribution behaviors, including extreme behavioral types—fully selfish (contribute zero), fully altruistic (fully contribute), or less extreme heuristics of “fair-sharing” (always contributing 50%), could explain the lack of treatment responses. For all of these, changes in cooperation incentives at the margin would not impact behavior and we would not observe treatment effects. Both the distributions of individuals’ contributions and expectations (Fig. 5) show that this is not the case, unveiling large heterogeneity (variance) in contributions both within and across samples (see also *SI Appendix*, Fig. S3 for the dispersion of individual contributions and individual expectations). For the different MPCR levels, there are no systematic differences in the distributions of individual contributions or in the expectations of others, both for the pooled data and within each sample (Fig. 5, Kolmogorov–Smirnov tests for equality of distributions show that none of the comparisons are statistically significant, all *P*-values > 0.05; see *SI Appendix*, section 3.1 for all *P*-values from KS tests). Across treatments, there are also no systematic significant differences in the distributions for deviations of individual expectations from individual contributions (*SI Appendix*, Fig. S4, all *P*-values > 0.05 from Kolmogorov–Smirnov tests, with the exception being the comparison of *LowMPCR* to *HighMPCR-LowE* in the pooled data, *P*-value = 0.03; see *SI Appendix*, section 3.1 for all *P*-values from KS tests). In summary, the average and the distributions of behavior do not significantly differ across variations in the marginal benefits from public good contributions, in all samples for contributions or expectations of others’ contributions.

### Expectations Are Closely Correlated with Cooperative Behavior, But Not Differently for Variations in Benefits from One-Time Cooperation.

Expectations positively and robustly predict contributions in all treatments and samples in a similar way. First, at the individual level, for all treatment conditions and samples, higher expected contributions of others correlate significantly with higher own contributions (between samples, point estimates of correlation range between 0.63 and 1.03 for estimated increases in contributions based on a unit increase in expected contributions by others; see *SI Appendix*, Table S7). Second, and importantly, the correlation between contributions and expectations is not significantly different when comparing the treatments with high and low MPCR (see nonsignificant interaction effects for both the pooled data and all samples separately in *SI Appendix*, Table S7). In summary, across samples, we find strong evidence of similar reciprocal behavior across variations in the benefits to contribute. Further, since patterns of expectations are similar across treatment conditions and participants’ contributions are strongly correlated with their expectations, they do not systematically or significantly respond to changes in the benefits from cooperation, displaying similar cooperation levels.

### Inattention, Randomness, or Confusion?

The results presented above do not appear to be motivated by general inattention nor confusion by participants that would result in random behavior, in line with recent evidence on confusion not explaining cooperative behavior in public good games (36). First, as opposed to purely random choices, the distributions of contributions and expectations are not uniformly distributed (that is, all *P*-values < 0.0001 from one-sided Kolmogorov–Smirnov tests against uniformly distributed random integer variates on the interval [0,100]). Second, only 2.8% of participants reported not having fully understood the decision task. And more importantly, the results do not change when excluding these participants.

## Results—Study 2

### Variations in Benefits from One-Time Cooperation in More Complex Group-to-group Public Good Settings Do Not Affect Cooperation Levels.

**Fig. 6.**
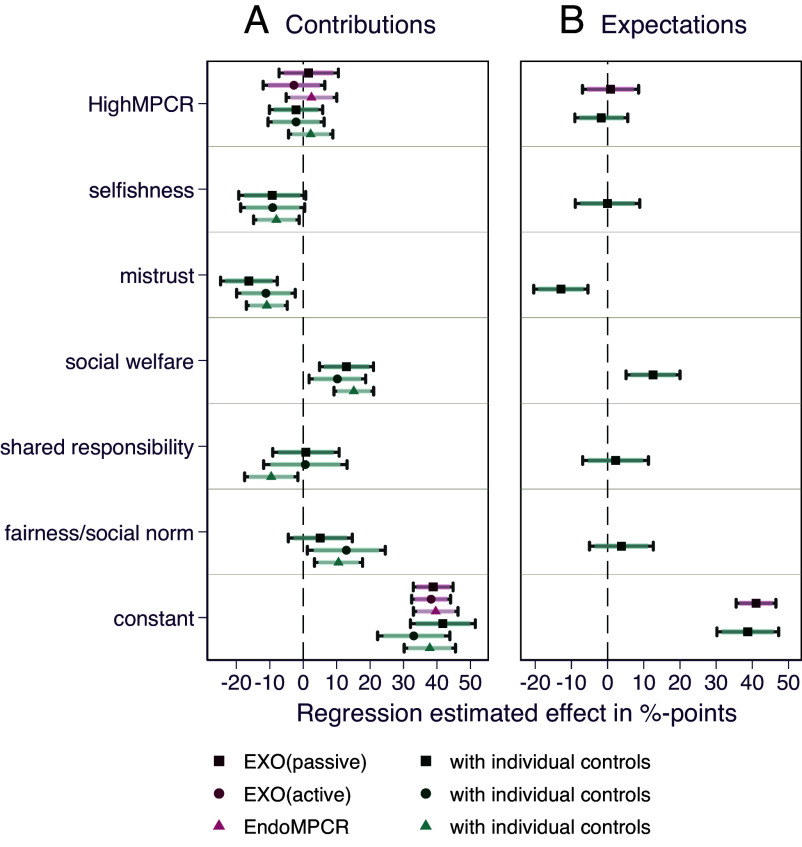
Point estimates and CI (90% CI indicated by shaded spikes, and 95% CI indicated by the caps at the ends of each side of the CI) from OLS regressions with robust SE for the main behavioral outcomes in Study 2: contribution to the public good in percentage of endowment (*A*) and individual expectations of the behavior of others (*B*). Models are estimated separately for the comparisons in *EXO(passive)* (n = 156), *EXO(active)* (n = 164) and EndoMPCR (n = 320). Explanatory variables in models without individual controls are the treatment dummy (*HighMPCR)* indicating the effect of the treatment condition as compared to the *LowMPCR* condition. In models with individual controls, we include self-reported motivations from postexperimental questionnaires. Constant indicates the mean of the respective dependent variable for the *LowMPCR* condition. Note that in *EndoMPCR* the “Low’” comparison category is groups with MPCR strictly lower than 0.8 and higher or equal to 0.4. 77.5% of groups endogenously established the highest possible MPCR of 0.8, and only 6.25% of groups did not increase the MPCR at all from 0.4. For this reason, we chose to group all those that did not increase the MPCR to the highest value of 0.8 together in *LowMPCR*, to ensure a large enough sample in the “low” category for statistical comparability. See *SI Appendix*, Tables S10 and S11 for the full regression outputs behind this figure.

The results for the one-time (single) decision public good setting of Study 1 extend to the more complex decision settings considered in Study 2. Recall, in Study 2, we examine one-time decision settings including providers and donors to public goods in two subgroups. We find that providers do not significantly increase cooperation for higher MPCRs (all *P*-values > 0.5; see Fig. 6 and *SI Appendix*, Tables S8 and S10), irrespective of whether the benefit from the public good was exogenously varied by the experimentalists with passive beneficiaries [Fig. 6*A*: *EXO(passive)*: ß = 1.64; *P*-value = 0.72; 95% CI: −7.23, 10.51] or with active beneficiaries sending donations in cash-transfers [Fig. 6*A*: *EXO(active):* ß = −2.78; *P*-value = 0.55; 95% CI: −11.99, 6.44] or when the benefits from the public good were endogenously defined through donations by beneficiaries [Fig. 6*A*: *EndoMPCR*: ß = 2.47; *P*-value = 0.52; 95% CI: −5.10, 10.03].

The coefficients for self-reported motivations to cooperate are, as for Study 1, consistent with the expected sign (Fig. 6*A* and *SI Appendix*, Table S10). The only exception is shared responsibility motivations, which under the presence of beneficiaries seems to have a more blurred effect [insignificant effect for *EXO(passive)*: ß = 0.82; *P*-value = 0.87; 95% CI: −9.1, 10.75; insignificant effect for *EXO(active)*: ß = 0.64; *P*-value = 0.92; 95% CI: −11.83, 13.11; and significant negative effect for *EndoMPCR*: ß = −9.56; *P*-value = 0.02; 95% CI: −17.52, −1.61; see Fig. 6*A* and *SI Appendix*, Table S10]. Also, while there are interaction effects of social welfare considerations with the high MPCR condition, these are not stable or consistent in sign across treatments [insignificant effect for *EXO(passive)*: ß = 5.30, *P*-value = 0.53; positive effect in *EXO(active)*: ß = 16.80, *P*-value = 0.05; negative effect in *EndoMPCR*: ß = −11.77, *P*-value = 0.09; see *SI Appendix*, Table S10]. Moreover, the same pattern of results holds relative to Study 1 in regard to: i) the role of expectations of others among providers (Fig. 6*B* and *SI Appendix*, Tables S9, S11, and S12), ii) the distribution of cooperation and expectations of cooperation among providers (*SI Appendix*, Fig. S6), and iii) the lack of support for results being driven by inattention, randomness, or confusion (*SI Appendix*, section 4.1).

## Discussion

Our results provide evidence on limits to the positive relationship between cooperation levels and incentives to cooperate in important naturally occurring one-time cooperation encounters. Aggregating over decision environments in Studies 1 and 2, we provide evidence for a lack of systematic responsiveness to large increases doubling the public good benefits in single-decision settings. The lack of a significant and consistent response to increasing benefits for cooperation holds across participant pools (UK general population vs. students), the physical distance between participants (online vs. in the laboratory), and more complex settings considering group-to-group interactions of providers and donors to public goods (passive vs. active beneficiaries). These results are in sharp contrast to previous literature, thus warning against simply extrapolating evidence to one-time (single-decision) cooperation encounters from public good studies with repeated interactions, situations where participants make multiple decisions across treatment parameters, or across different possible cooperation levels of others in their groups, or PD game settings.

These results shed light on the fundamentals of cooperative behavior. In particular, we believe our results contribute to the advancement of understanding the necessary and sufficient conditions that trigger behavioral responses to changes in (economic) incentives, including changes in the marginal benefits from cooperation. First, we identify, through the mediating role of beliefs of others, a mechanism to explain the lack of significant increases in cooperation to increases in the marginal benefits from public good contributions. Participants’ cooperative behavior in our studies is highly correlated with their expectations of others’ cooperativeness. This is in line with a main motivation for cooperation in public good settings identified as the social norm of (anticipated) reciprocity (37, 38). From a behavioral perspective, individuals who are motivated by reciprocal preferences will cooperate more with higher benefits from cooperation if they also expect others to do so (29). Importantly, in the one-time (single) decision environment we consider, expectations of others’ behavior remain stable across changes in the benefits from cooperation and the correlation between own expectations and own contributions does not vary across individuals facing different marginal benefits from cooperation. Therefore, cooperation levels are not found to vary with the benefits from cooperation.

Second, the impaired possibility to activate forward-looking strategies in single-decision environments may be an additional reason for the lack of responses to the increases in the benefits from cooperation that we report. Previous research considering repeated interactions has proposed long-term payoff considerations as motivations for cooperation in public good environments (10, 39). Specifically, to explain the mechanism through which higher benefits from cooperation can induce higher cooperation levels, Isaac et al. (10) developed a theoretical model for expected intertemporal gains from cooperation where individuals are forward-looking and perceive their contributions as meaningful signals to other members of their group. In the one-time (single) provision environment an individual’s contribution does not have signal value as there are no future expected joint contributions. Further support for this argument comes from taking a closer look at the behavior of beneficiaries and providers in the *EndoMPCR* treatment of Study 2, where the value of the MPCR depends on the level of donations. The beneficiaries make their donation decisions before the public good providers make their decisions. As such, beneficiaries can be forward-looking, and we find that indeed they use the opportunity to do so. In fact, the majority of beneficiary subgroups (62 out of 80 groups, 77.5%) voluntarily invested the sufficient amount to guarantee the high benefit from the public good, and thereby potentially enhancing the welfare of the whole group (see description of Fig. 6 and *SI Appendix*, section 2). On the contrary, the public good providers, who move last, cannot be forward-looking, and we observe that they did not respond on average to the higher gains from cooperation.

The results presented here provide a critical complement to prior research on cooperation by focusing on settings that capture important real-life situations for public goods provision such as individuals’ responses to immediate disaster relief efforts. Previous literature provides a thoughtful discussion of the psychological motivations for why prosocial behavior emerges in response to disasters (40, 41). Included in those motivations are shared social identification, such as an expectation of support from others or convergence behavior where those unaffected choose to give support, a form of reciprocal or empathy altruism. While the authors in (40) discuss that some individuals may determine their degree of prosocial behavior based on a *cost-reward analysis* for helping others, interestingly, there is evidence from interviews that at least some volunteers in emergency situations ignored the potential cost/personal risk of their involvement (41, page 51). These findings are consistent with our result that one-time cooperation decisions may not reflect differences in the cost/benefits of the public goods provided. Thus, our findings suggest that in distinct one-time (single) decision cooperation settings, individuals may not evaluate the social dilemma differently across differences in the benefits derived from cooperation. This is despite lower conflicting interests between individual and collective welfare with higher benefits from public goods. Further, the average cooperation that we observe across treatments, while positive, is closer to previously reported cooperation levels for low benefits from cooperation (MPCRs = 0.3 to 0.4). Based on the postdecision survey, the fact that the motivation to increase social welfare is neither more frequently mentioned in the treatments with high benefits from cooperation, nor is it found to be a stronger driver of cooperation, suggests that individuals who are (or are not) social-welfare oriented are so irrespective of the benefits from one-time cooperation. While there might be evolutionary advantages for this behavior, these results raise questions as to why cooperation levels remain relatively low in the high benefit environments, including whether cooperation decisions in such settings are based on more than just cost/benefit ratios, such as emotional responses.

Finally, our results open broad avenues for future research building upon the important role of beliefs of others’ cooperation as a strong determinate of cooperativeness. In one-time encounters, the success of public good provision depends on the willingness of individuals to cooperate even under limited to no information on others’ preferences, less prevalent reputational concerns, and when long-term relationships are not salient. In this light, it seems vital to advance our understanding on how individuals process information regarding the value of their contribution to one-time public good provision, and how this translates into shaping their expectations on the strength of group support.

## Materials and Methods

### Procedures.

The experiments were programmed in oTree (42). Informed consent was obtained for experimentation with human subjects, and the experiments (as part of a broader research agenda) received IRB approval from the Board for Ethical Questions in Science of the University of Innsbruck (14/2022). The general population sample in Study 1 and Study 2 was recruited using Prolific with participants from the U.K. and requirements that they were fluent in English and had a minimum approval rate of 95% from previous studies. The student population samples in Study 1 were recruited from the subject pool of the EconLab of the University of Innsbruck. Participants were recruited into sessions that randomly administered one of the treatment conditions in a between-subject design. Participants were informed that their earnings were conditional on completing the study and that they would be timed out after 90 min. Participants could only participate once. At the beginning of all experiments (within Study 1 and within Study 2), all participants received the same (treatment specific) instructions, numerical examples, and were asked to answer a series of comprehension questions. Two of the comprehension questions required participants to type numerical responses regarding the private value of an Experimental Currency Unit (ECU) and the MPCR (“*Each ECU a participant moves to the Group Account reduces the value of his/her Private Account by ECUs*:*___*” and “*Each ECU a participant moves to the Group Account generates earnings from the Group Account for each member of his/her group of ECUs:___*”). In the *EndoMPCR* treatment of Study 2, participants had to answer, *“The Group Account earnings from each ECU a Provider allocates to the Group Account depend on the sum of allocations in the Transfer Account by the Beneficiary members of his/her group—True/False*”. All participants had to answer the comprehension questions correctly before they could move forward in the experiment. In Study 2, participants only found out after the comprehension questions whether they were randomly allocated to the “Provider” or “Beneficiary” role. Thereafter, each participant in Study 1 and all of those assigned to the Provider role in Study 2 took part in three tasks: 1) an incentivized estimation of the expected behavior of the other members with whom they would be grouped to calculate payoffs, 2) their individual contribution to the public good, and 3) a questionnaire containing questions about the main motivations for their decisions and about their donation and volunteering in their day-to-day lives. To incentivize informed estimates, participants could earn (£/€)1.5 divided by the absolute difference between the actual value and their estimate, up to a maximum of (£/€)1.5. Participants were informed in the instructions that they would be asked to give this estimate before decision-making. Across all treatments in Study 1 and Study 2, participants earned on average £ 5.20 for UK general population and € 5.35 for Austrian students, which included a base payment of (£/€) 2, and average incentives of (£/€) 3.28 whereby 100 ECUs = 2(£/€).The experimental session lasted on average 20 min. See details in *SI Appendix*, section 2.

### Preregistrations.

Study 2 was preregistered first (https://aspredicted.org/C6T_2FL). The unexpected lack of significant statistical differences in Study 2 in response to different MPCR treatments in the provider-beneficiary framework motivated the investigations of Study 1 in this paper. Study 1 entailed, first, conducting additional experiments in Prolific for the general population sample (preregistration: https://aspredicted.org/HSX_P32). These results motivated collecting the additional data with students at the University of Innsbruck, both online and in the economic laboratory (preregistration: https://aspredicted.org/82N_8DJ). Finally, the persistence of these results motivated the final robustness experiments with Prolific reversed order contribution and expectation decisions (preregistration: https://aspredicted.org/SD2_L1N).

### Statistical Power and Minimum Detectable Effect Sizes.

We designed our studies to be able to detect meaningful effect sizes smaller than those reported in previous literature reviews and meta-analyses analyzing changes in the MPCR in public goods games for similar group sizes and MPCR levels (23–25). The reported sample sizes for each subsample in Study 1 and the full sample in Study 2 were calibrated to have an 80% power to detect a 15%-point treatment difference between conditions (with conventional *P*-values of 5%). For comparability reasons, we kept the sample size per treatment comparison in each subsample for Study 1 similar to that in Study 2 (which as discussed above was conducted first) as estimated in that power analysis. The a priori power analysis was conducted using the *power* command in STATA for two-sample means tests (two-sample *t* tests), with calculations based on data from Blanco et al. (35) (first-period with providers and beneficiaries making decisions), entailing a mean contribution of 28.46% of endowments and SD of 31.29% of endowments with four public good providers in a group. In *SI Appendix*, Tables S1, S2, S8, and S9, we report all *P*-values from two-sample *t* tests for all comparisons of means in Studies 1 and 2. Note that these are equivalent to the *P*-values from the preregistered OLS regressions reported in Figs. 4 and 6 (model without individual controls).

Pooling the data of Study 1 for the main comparisons (Fig. 3), our sample size of a total of n = 716 independent observations allows us to exclude small minimum detectable effect sizes (following 43) of 8.19%-points and 8.04%-points for comparing *LowMPCR* with *HighMPCR* and *HighMPCR-LowE*, respectively. For Study 2, based on a total sample of n = 640 providers, the minimum detectable effect sizes are 12.7%-points (n = 156), 13.2%-points (n = 164), and 10.9%-points (n = 320) for the *EXO(passive)*, *EXO(active),* and *EndoMPCR* comparisons, respectively.

To illustrate, previous studies report first-period differences of 17%-points difference between MPCRs of 0.3 and 0.75 with adjusted endowments, as in our *HighMPCR-LowE* condition (8) and of 30%-points and 20%-points, for changes in MPCR from 0.3 to 0.75 and 0.6, respectively, holding endowments constant (15, 17). Studies using a within-subjects design for changes in the MPCR and strangers-matching protocols report 20%-points differences for groups of size 3 and MPCR values of 0.4 and 0.8 (11, 20). Finally, even with a repeated-strangers-matching protocol, van den Berg et al. (22) present evidence for first-round differences of 30% between their lowest (0.367) and highest (0.833) MPCR conditions, while also holding constant endowments between treatments. A study conducted after our preregistrations reports a smaller effect size than those reported by previous studies, down to 10%-points difference between their low and high MPCR conditions (0.4 and 0.8, respectively, for groups of size 3) (29).

In summary, we can exclude smaller effect sizes to those reported in the previous literature, and which are small in absolute terms (as low as 8%-points for Study 1) considering the large increase of 100% in the marginal benefits from cooperation in moving from an MPCR of 0.4 to one of 0.8.

## Supplementary Material

Appendix 01 (PDF)

## Data Availability

All data (anonymized), analysis files, anonymized preregistrations, experimental instructions and survey questionnaires have been deposited in Open Science Framework (https://osf.io/w5q2t/) (44).
