# Supplementary material for: Increasing benefits in one-time public goods does not promote cooperation
Source: Proc Natl Acad Sci U S A. 2024 Oct 4;121(41):e2410326121. doi: 10.1073/pnas.2410326121 (PMC11474087; doi:10.1073/pnas.2410326121)
Supplement: Supplementary file 1 — Appendix 01 (PDF) [file pnas.2410326121.sapp.pdf]

PNAS

Supporting Information for

Increasing benefits in one-time public goods does not promote cooperation

Natalie Struwe [a], Esther Blanco [a,b], James M. Walker * [b,c]

[a] Department of Public Finance, University of Innsbruck, Universitaetsstrasse 15, 6020 Innsbruck, Austria.

[b] The Ostrom Workshop, Indiana University, USA

[c] Department of Economics, Indiana University, Wylie Hall 105, Bloomington, IN 47405, USA

* James M. Walker

Email: walkerj@iu.edu

This PDF file includes:

1. Extended Literature Discussion
2. Details on Experimental Design and Procedures
3. Additional Analysis Study 1 (Tables S1 – S7, Figures S1 – S4)
   3.1. Tests for equality of distributions in Study 1 (Figure S5)
   3.2. Results and discussion of check of assumptions to use linear regression models (OLS)
4. Additional Analysis Study 2 (Tables S8 – S12, Figures S6 – S7)
   4.1. Inattention, Randomness and Confusion in Study 2
5. Robustness Analysis of Treatments with reversed order of decisions (Tables S13 – S16, Figures S8 – S9)
6. Multiple Hypothesis Testing (Table S17)
7. Post-experimental questionnaire (survey questions) Study 1 and Study 2
8. SI References

Please find all experimental instructions and pre-registrations in the OSF repository:

osf.io/w5q2t

Please note that all data and analysis scripts will be made publicly available in this repository upon publication.

1. Extended Literature Discussion

The first study to test the relationship between benefits from repeated public goods and public good provision levels was Isaac et al. (1) who provided an operationalization of the voluntary contribution mechanism (VCM) and an experimental test of the theory of free-riding, considering group sizes of 4 and 10, and MPCR levels of 0.3 and 0.75. The authors' research showed that neither strong nor weak free-riding theories could successfully predict the observed behavior. Their research demonstrated that free-riding behavior was strongly influenced by repetition, as well as the MPCR (and group size). This seminal contribution triggered a large literature to further investigate the responsiveness of cooperation to the relative costs and benefits from public goods (for reviews see 2–4).

A major focus in this literature examines group size and MPCR effects on public good contributions (5–11). A positive MPCR effect has also been identified for children aged 6-12 years old (12). These studies all use finitely repeated public goods settings, although the positive MPCR effect has also been shown for infinitely repeated public goods (13). As opposed to repeated interactions in partner-matching, studies have also considered stranger-matching protocols (14, 15), where the former considered further group matching rules whereby groups were sorted based on ranking in previous contributions within a session.

Both Goeree et al. (6) and Goeschl et al. (10) used a within-subject design to test subjects' responses to 10 variations of MPCRs with no feedback between decisions. These designs are often referred to as "one-shot" decisions, although a within-subjects design differs fundamentally from the true one-shot (one-decision) setting we consider, as subjects in within-designs can strategically compare different levels of the MPCR.

Another strand of the literature has considered heterogeneous MPCRs within groups (16–21), highlighting that those subjects with a high MPCR contributed more than those with lower MPCRs, but the heterogeneity in MPCRs could have negative effects on aggregate public good provision levels.

Further, some studies consider the effectiveness of sanctioning institutions such as peer-punishment coupled with different MPCR levels (7, 19, 20) or a tax-subsidy scheme whereby deviations from the average contributions in one's group are either rewarded or penalized (22). More recently, Jin et al. (23) consider the effect of MPCRs on institutional choices, allowing participants to vote for peer punishment, central punishment or verbal *gossip* and ostracism. They find that while the MPCR affects contributions, it does not affect the probability of voting for the sanctioning institutions.

Finally, the positive MPCR effect has also been found in appropriation frames, as opposed to the public good provision frames considered in the references above (24–26).

In a recent and closely related online experiment run via Amazon Mechanical Turk (MTurk), van den Berg et al. (15) examined the effect of incremental changes in the MPCR on public good provision in a between-subjects design. While the authors refer to this as one-shot decisions, participants made repeated decisions in randomly re-matched groups of three (created from groups of size 18). The authors find that, on average, contributions increase substantially for MPCRs, from below 40% for an MPCR of 0.4 to almost 70% of endowments for an MPCR of 0.7. Their results are qualitatively stable when considering only the decisions from the first round where participants know that they will be making decision in multiple decision rounds. Although this study does not examine a setting where subjects interact in a truly one-time (single) decision setting, it provides an example of how repetition (even with random re-matching) may influence participant's strategic thinking in a way that may be influenced by norms of reciprocity and forward-looking behavior.

The experiment of Gächter & Marino Fages (27) was run via Prolific, examining between-subject changes of different MPCRs. The authors used a two-stage design where participants first chose conditional contributions via the strategy method, that is, decide on a contribution for each of the possible average contributions of the other group members. The participants then made a contribution decision in a one-shot public good game. The authors find that both conditional (part 1) and direct-response (part 2) contributions are higher with the higher MPCR, with a 10%-points difference between their low and high MPCR conditions (0.4 and 0.8, respectively, for groups of size 3) in part 2. Notice that while part 2 is indeed equivalent to the decision that participants in our experimental setting make, in our study participants do not face a part 1 where they reflect on their decisions given all potential scenarios of the other group member's behavior. The level of average *expectations* regarding the behavior of others in Gächter & Marino Fages from their Prolific sample is approximately 40% of endowments for the low (0.4) MPCR and 46% for the high (0.8) MPCR. This 6%-points difference is statistically significant in their study. In our Prolific sample, average expectations are 42% (*LowMPCR*), 41% (*HighMPCR*) and 39% (*HighMPCR-LowE*) with the differences not being statistically significant. Note, for our sample, the levels of these expectations regarding the contributions of others are all closer to the expectations for the low MPCR in Gächter & Marino Fages.

Further, there is recent experimental evidence on non-repeated multi-level public goods where participants can compare across public goods with different MPCRs. Gallier et al. (28) provide evidence from an experiment where participants from different regions and neighborhoods decided on contributions to a local (including only members from same neighborhood) vs a regional (including members from the same metropolitan area) public good. Increasing the MPCR to the regional public good (while keeping constant the MPCR for the local public good) resulted in a substitution effect between both public goods and no overall increase in efficiency. That is, the authors observe an increase in contributions to the regional public good, but at the

expense of contributions to the local one. Catola et al. (29) confirm such a substitution effect in their online experiment conducted in Prolific.

Related evidence exists from studies considering non-repeated Prisoners' Dilemmas with changes in the monetary payoffs from mutual cooperation, or similarly, the benefit-to-cost ratios. For example, Charness et al. (30) show in their between-subjects comparison that higher payoffs for cooperation significantly increase cooperation rates. Further, in their simultaneous play modified two-person PD with continuous transfers between 0 and 10 tokens, Capraro et al. (31) find that a substantial number of participants transfer 50% of their $.10 endowment across treatments. In their game, each $.01 transfer is multiplied a by a constant, of value between 2 and 10, varying the benefit-to-cost ratio of the transfer. Further, they find a shift towards less 0% transfers and more full (100%) transfers for higher benefits. Gupta et al. (32) provide a direct comparison of behavior in a laboratory sample (University of Pittsburg) and two online samples (Mturk and Prolific) in Prisoners' Dilemmas varying the marginal incentives to cooperate, similar to the experimental design of (30), but with a within-subject comparison. As opposed to the laboratory sample, the response of Prolific participants to changes in marginal cooperation incentives is "near negligible". Finally, in a recent study, Gächter et al. (33) consider orthogonal variations of payoffs from cooperation in a one-shot (non-repeated) Prisoner's Dilemma for both within- and between-subject comparisons. The main finding is that cooperation increases with increasing benefits from mutual cooperation (as compared to mutual defection).

Our setting with *endogenous* variations in the MPCR (*EndoMPCR* treatment of Study 2) also has similarities to public good games where the production of public goods depends endogenously on group effort. These include threshold public good games, where public good providers receive the benefits from public goods only if provision meets or exceeds a pre-defined threshold (referred to as *provision points*) (see, for example, 16, 34–38). Croson & Marks (39) have established the term *step return* in threshold public goods, to capture the idea that the MPCR depends on the discontinuity defined by the thresholds. They find that with increasing step return requirements, contributions to the public good significantly increase. Similarly, in probabilistic public good games, where the probability of provision of the public good increases with contributions, the expected value of the MPCR depends on the expectations of the groups' efforts (24, 40–42). The decision setting *EndoMPCR* considered in our study differs from these studies in the sense that there is no minimum contribution required or probability attached to produce the public good. The level of public good provision increases with the level of contributions. However, the marginal benefit (MPCR) increases with increasing transfers. Finally, the case of continuous endogenous increases of the MPCR is also related to a study by Noussair & Soo (43) considering a *dynamic public good* where the value of the MPCR depends on a group's contributions from the previous period. They find

significant heterogeneity in group dynamics, where 30-40% of the groups yield contributions that are sufficiently high so that the MPCR increases over time, close to the maximum possible.

2. Details on Experimental Designs, Behavioral conjectures & Experimental procedures

As discussed in the paper, Study 2 was conducted prior to Study 1, with results from Study 2 motivating Study 1. Both Study 1 and Study 2 were between-subject conditions where participants were presented only with one treatment condition and participants from Study 2 were excluded to participate in Study 1.

Experimental Design – Study 1. The objective of Study 1 was to provide, in one-time (single) decision environment, a relatively simple test for the responsiveness of cooperation to changes in the MPCR when subjects have a continuous choice in cooperation. The VCM environment was chosen based on the breadth of its use in experimental studies on public good provision. A group consists of $n = 4$ members, where each member receives an endowment of w that can be used to make contributions $g_i \in [0, w]$ to a Group Account of size $G = \sum_{i=1}^{n_i} g_i$ or a Private Account (with a return of 1 per unit contribution)The Group Account constitutes a public good with an equal marginal return (MPCR) of a for all group members, where $\frac{1}{n} < a < 1$, so that the cumulative value of a contribution across all group members exceeds the marginal cost of a contribution. The payoff function for each group member is given by equation (1).

$$\pi_i = w - g_i + aG \qquad (1)$$

We implement three different treatment conditions, varying the level of the parameter a and the individual endowment of the group members. In *LowMPCR*, $a_{low} = 0.4$; in both *HighMPCR* and *HighMPCR-LowE,* $a_{high} = 0.8$. Based on findings of van den Berg et al. (15), we chose the values of the MPCR of 0.4 for the low condition and 0.8 for the high conditions to allow for a substantial amount of variation in behavior. Between *LowMPCR* and *HighMPCR* we hold constant the endowment $w = 100$ ECUs (Experimental Currency Units). This implies a potential wealth effect at the efficient outcome in *HighMPCR* compared to the *LowMPCR* condition, as the maximum (social optimum) payoff in *HighMPCR* is 320 ECUs for each participant and in the *LowMPCR* it is 160 ECUs. To account for this effect from changing the MPCR (a treatment change first examined in Isaac et al. (1), we also implement *HighMPCR-LowE* where all participants receive an endowment of $w = 50$ ECUs, thus holding constant the maximum payoff as compared to *LowMPCR.*

In all treatments, contributions to the public good constitute a social dilemma in each condition and free-riding incentives exist ($a < 1$). Following the *symmetric binary choice approach* as

developed for linear public goods games (5), one can compute the monetary gains to an individual from cooperation comparing either the social optimum outcome (all members contribute fully to the public good) to the Nash Equilibrium outcome assuming fully self-regarded, payoff-maximizing individuals (where all members contribute nothing to the public good), given by equation (2):

$$\pi^{SO} - \pi^{NE} = anw - w = w(an - 1) \qquad (2)$$

With *n*=4, the numerical values for the three different MPCR conditions are 60 for *LowMPCR*, 220 for *HighMPCR*, and 110 for the *HighMPCR-LowE*, showing that the gains from full cooperation are substantially higher with the MPCR of 0.8 than with the MPCR of 0.4, in both high MPCR treatments. In summary, by design, increasing the MPCR in linear public goods games increases the gains from cooperation. Note further, holding the return from the private good constant, increasing the MPCR also reduces the opportunity cost of making a contribution.

Our pre-registered Conjecture 1 below is based on these observations and two primary results from the previous literature: (i) a well-established positive relationship between contribution levels and the MPCR in previous experimental studies (see references in the introduction), and (ii) evidence that average contributions in one-shot public good games (holding constant the MPCR) are not significantly different for online vs lab settings (e.g., 44, 45).

> ***Conjecture 1****:* In all samples, average contributions (in percent of endowment) to the public good will be higher for high MPCR environments of 0.8 (*HighMPCR* and *High MPCR-owE*) compared to the low MPCR environment of 0.4.

Experimental Design – Study 2. In the provider-beneficiary decision setting, a group consists of 8 members in two subgroups, $n_I = 4$ providers and $n_O = 4$ beneficiaries. Both providers and beneficiaries receive an endowment of $w = 100\,ECUs$. Providers can make contributions g_i out of endowment w, with $g_i \in [0, 100]$ to a Group Account $\mathrm{G} = \sum_{\mathrm{i}=1}^{\mathrm{n_i}} \mathrm{g_i}$ that constitutes a public good with an equal marginal return (MPCR) of a for providers and beneficiaries, where $\frac{1}{(n_I + n_O)} < a < 1$. Beneficiaries cannot make contributions but benefit from public good provision, where the decision faced by beneficiaries varies across treatment conditions.

In the treatments *EXO(passive)-LowMPCR* and *EXO(passive)-HighMPCR*, beneficiaries are inactive, they simply receive the benefit from contributions to the Group Account by providers. The Payoff functions for individual providers and beneficiaries respectively are as follows:

$$\pi_i = w_I - g_i + aG \qquad (3)$$

$$\pi_o = w_O + aG \quad (4)$$

In *EXO(passive)-LowMPCR* the MPCR is exogenously defined at $a_{low} = 0.4$, while it is exogenously defined at $a_{high} = 0.8$ in *EXO(passive)-HighMPCR*.

In all treatments with active beneficiaries, beneficiaries can use their endowment to make a donation, as a transfer $t_j \in [0, w]$ to a Transfer Account of size $T = \sum_{j=1}^{n_o} t_j$. Importantly, beneficiaries make their transfer decisions before providers make their contribution decisions. The use of the funds in the Transfer Account varies between treatments. First, in *EXO(active)-LowMPCR* and *EXO(active)-HighMPCR* donations entail cash-transfers to providers, whereby the Transfer Account is shared equally among providers. The payoff functions are defined in equations (5) for individual providers and (6) for individual beneficiaries:

$$\pi_i = w_I - g_i + aG + \frac{1}{n}T \quad (5)$$

$$\pi_o = w_O - t_j + aG \quad (6)$$

In *EXO(active)-LowMPCR* the MPCR is exogenously defined at $a_{low} = 0.4$, while it is exogenously defined at $a_{high} = 0.8$ in *EXO(active)-HighMPCR*.

In the *EndoMPCR* treatment, the donations to the Transfer Account are used to define the MPCR of the public good, from the starting value of 0.4 to a maximum value of 0.8. Two conditions discussed below allowed beneficiaries to endogenously increase the MPCR through transfers. In this paper, we only consider provider's responses to the MPCR (irrespective of the specific technology used to endogenously increase the MPCR), therefore we pooled the data of both conditions for endogenous MPCRs in the analysis and results presented. We outline in the following the functional forms of how beneficiarys' transfers affected the MPCR. In case (1) the MPCR is increased from the starting value of 0.4 to the value of 0.8 if beneficiaries' transfers meet a publicly specified threshold. If transfers offered fall short of the threshold, they are returned to beneficiaries (a "money-back guarantee" (34)). Further, transfers above the threshold are refunded in proportion to individual transfers offered (46–48). As can be seen in equation (7), the MPCR is now a function of the sum of transfers offered by beneficiaries, $a(T)$:

$$a(T)_{Thres} = \begin{cases} 0.8 & if\ T \geq 25\%\ of\ beneficaries'\ group\ endowment \\ 0.4 & if\ T < 25\%\ of\ beneficiaries'\ group\ endowment \end{cases} \quad (7)$$

The value of the threshold is chosen such that it corresponds to the average transfers offered in the first period with transfers in Blanco et al. (49), where beneficiaries offered 23% of their group endowment in transfers in the *Equal* treatment condition.

In case (2) the MPCR continuously increases within the interval of $[0.4\,,0.8]$ for any transfers between 0% and 25% of beneficiary group endowment. More specifically,

$$a(T)_{Cont} = \begin{cases} 0.8 & if\ T \geq 25\%\ of\ beneficiaries'\ group\ endowment \\ 0.4 + 0.4\left(\frac{T}{100}\right) & if\ T < 25\%\ \ of\ beneficiaries'\ group\ endowment \end{cases} \qquad 8)$$

This implies that the MPCR can never be below 0.4 nor above 0.8. With the Transfer Account reaching 25% of beneficiary group endowment, the highest MPCR of 0.8 is reached for the same level of transfers (i.e. same investment by beneficiaries) as in the threshold case above. Transfers above the necessary investment to reach the highest MPCR of 0.8 are refunded to the beneficiaries in proportion to their individual transfers offered.

The payoff functions for individuals are defined in equations (9) for providers and (10) for beneficiaries:

$$\pi_i = w_I - g_i + a(T)G \qquad (9)$$

$$\pi_o = w_O - t_j + a(T)G \qquad (10)$$

For Study 2, the pre-registered conjectures follow:

> ***Conjecture 2****: Average contributions in EXO(passive)-HighMPCR will be significantly higher than average contributions in EXO(passive)-LowMPCR.*
>
> ***Conjecture 3****: Average contributions in EXO(active)-HighMPCR will be significantly higher than in EXO(active)-LowMPCR.*

Additional hypotheses reported in https://aspredicted.org/C6T_2FL referred to the comparison of endogenous vs. exogenous imposition of the MPCRs. Given the non-responsiveness to variations in the MPCR observed in our data, these other hypotheses have become of secondary importance.

Belief elicitation Study 1 and Study 2. The belief elicitation reported in the manuscript entailed asking participants to estimate in integer values the average amount of ECUs they expected each of the other participants in their group to contribute to the Group Account. This referred to all treatments in Study 1 and to treatments *EXO(passive)-LowMPCR* and *EXO(passive)-HighMPCR* of Study 2. Beliefs were elicited before own contribution decisions. This procedure provided further assurance that participants thought carefully about how others might interpret the decision opportunities within this particular decision environment and to encourage behavior motivated by expected conditional reciprocity to unfold (given the lack of

repeated interactions). Notice that existing studies by Croson (50, 51) concerned with the effect of eliciting beliefs of others on own contributions in repeated versions of the public good game with partner matching protocols find that when beliefs are elicited before decision making contributions are lower than in a control of no belief elicitation. Notice though, we are not primarily interested in the level of contributions to the public goods for a given treatment, but in the difference in the level of contributions between treatments across different MPCRs. Because we hold constant the order of belief elicitation and contribution decisions for variations in the MPCR, our protocol might impact all observed contribution levels, but not have a differential impact on contributions to the public good across our different MPCR treatments (Table S7 provides support by the non-significant interaction effects between treatments and expectations on individual contributions). Further, for the purpose of examining the robustness of our results, we collected additional data via Prolific (*rob* treatments) for each of the three MPCR treatments. For some of the more complex treatments in Study 2 the belief elicitation task was also more complex (providers' beliefs about beneficiaries transfers and vice-versa), and thus are not reported here as do not refer to the expected behavior of peer-providers to the public good.

Procedures prolific online experiment Study 1. Data collection via Prolific took place online in October 2022. A total of 232 participants were recruited in three sessions on two consecutive days. Participants were recruited from the U.K. Prolific population with the requirements that they were fluent in English and had a minimum approval rate of 95% from previous studies. The session was open until the necessary number of participants that finished the experiment was reached. Groups were matched into a subgroup of four according to the arrival time of participants to the waiting page after the control questions. Thereafter, the sum of decisions made by four participants in a matched group was calculated to determine individual payoffs that were sent to participants as bonus rewards via Prolific. The experiment was designed to take about 20 minutes and participants who took longer than 90 minutes were timed out via Prolific automatically. Participants earned on average £5, which included a base payment of £2. Participants could only participate once.

The data collection for the robustness treatments for reverse beliefs elicitation took place via Prolific on May 17th, 2023 with a total of 236 participants. Participants first made a contribution decision and then were confronted with the (previously unmentioned) incentivized expectation task. In these treatments, to reduce waiting time for participants, we deleted the waiting-pages after control questions and matched participants into groups of four ex-post, based on their registration time (the time when they entered the experiment). Because matching into groups is only relevant to calculate payments and did not affect the experience of participants, differences in this technical detail of the procedures cannot impact individual decisions. All other procedures related to our initial experiments were held constant. One participant only

gave partial data and therefore this participant and their matched group members had to be dropped from the final sample. Average earnings were £4.80.

Procedures student online experiment Study 1. For the online experiments with the university students, participants from the student subject pool of the EconLab of the University of Innsbruck were recruited into simultaneously running sessions during March 2023 (using hroot (52)). On the day of the experiment, they received a participant-specific link that allowed them to participate only once, in a pre-specified time window. Participants were made aware that once they started the experiment, they needed to finish it within 90 minutes, or otherwise would be timed out (to follow the same protocol as in Prolific). The matching of four participants into groups was based on their registration time to sign up for the experiment. Payments were made online via PayPal. On average, participants earned 5.30€, including a base payment of 2€.

Procedures student laboratory experiment Study 1. For the laboratory sessions, participants from the student subject pool of the EconLab of the University of Innsbruck were recruited into 12 sessions during March of 2023 (using hroot (52)). Upon arrival, participants were randomly seated in the lab and instructed to proceed through the experiment on the computer. Instructions were not read out loud in order to follow the same protocol as in the online experiments. Participants could raise their hand to ask questions which were answered privately by the experimenter. The instructions included the sentence "All 4 members of a group will receive the same instructions." Both Isaac & Walker (11) and Isaac et al. (5) did not read out instructions aloud. After all participants finished the experiment, they were matched into groups of four based on their randomly assigned seat number. Payments were then made in cash. On average, participants earned 5.40€, including a base payment of 2€.

Procedures experiments in Study 2. Data was collected online during May 2022. Participants were recruited via Prolific. A total of 1280 participants were recruited on nine experiment days, resulting in eighteen sessions. Participants were recruited from the U.K. with the requirements that they were fluent in English and had a minimum approval rate of 95% from previous studies. Participants could only participate once. No participant of Study 1 had participated in Study 2. Participation took on average 20 minutes and participants earned on average £5.71, which included a base payment of £2.5. For each treatment, we recruited participants for two consecutive sessions, where recruitment for the second session only started after the first session had finished. Those participants who signed up for the first session made decisions in the role of beneficiaries and received instructions based on the treatment conditions to which they were assigned. Participants learned their type (beneficiary or provider, referred to as Type 1 or Type 2) only after answering all comprehension questions correctly. Groups of providers and beneficiaries were matched according to the arrival time of

participants to the waiting page. Each individual made decisions independently of the others in their session. The session was open until the necessary number of participants for the beneficiary session was reached. Then, the sum of decision taken by four beneficiaries in a matched group was calculated to determine the size of the Transfer Account for each group. The next session began immediately after the beneficiary session was completed. In this session, participants made decisions as providers receiving the relevant instructions for their treatment condition. They observed the aggregate decisions of the 4 beneficiaries with whom they were matched in the treatment condition and made decisions on provision to the public good. At the end of the providers' session, payoffs were calculated for each participant and sent to them as bonus rewards via Prolific.

3. Study 1 - Additional Analysis

Table S1. Contributions in Study 1: Summary statistics of average individual contributions in Study 1, in % of endowments, by treatment and sample. Std. deviations in parentheses. For comparison of contributions in *LowMPCR* with *HighMPCR* and respectively with *HighMPCRLowE* we report p-values from two-sample t-tests for the pooled sample and for each sample separately. P-values for the Pooled sample are 0.82 and 0.099. P-values for the General Population sample are 0.68 and 0.55. P-values for the student online sample are 0.93 and 0.097. P-values for the student laboratory sample are 0.42 and 0.57. In summary, none of these differences are significant at a conventional p-value < 0.05.

	**Pooled**	*n*	**GP**	*n*	**Students [online]**	*n*	**Students [lab]**	*n*
**LowMPCR**	**42.28** *(30.60)*	244	**39.58** *(30.05)*	80	**41.9** *(27.66)*	80	**45.23** *(33.74)*	84
**HighMPCR**	**41.63** *(33.46)*	240	**41.57** *(29.48)*	76	**42.36** *(36.91)*	80	**41** *(33.77)*	84
**HighMPCR-LowE**	**47.01** *(31.80)*	232	**42.32** *(26.12)*	76	**50.38** *(35.99)*	80	**48.16** *(32.14)*	76

Table S2. Expectations in Study 1: Summary statistics of average individual expectations in Study 1, in % of endowments, by treatment and sample. Std. deviations in parentheses. Note: For comparison of expectations in *LowMPCR* with *HighMPCR* and respectively with *HighMPCRLowE* we report p-values from two-sample t-tests for each sample separately. P-values for the Pooled sample are 0.6 and 0.47. P-values for the General Population sample 0.80 and 0.48. P-values for the student online are 0.33 and 0.18. P-values for the student laboratory sample are 0.14 and 0.66. In summary, none of these differences are significant at a conventional p-value < 0.05.

	**Pooled**	*n*	**GP**	*n*	**Students [online]**	*n*	**Students [lab]**	*n*
**LowMPCR**	**44.48** *(23.43)*	244	**42** *(22.61)*	80	**43.75** *(20.02)*	80	**47.55** *(26.89)*	84
**HighMPCR**	**43.31** *(26.38)*	240	**41.03** *(24.65)*	76	**47.45** *(27.28)*	80	**41.42** *(26.87)*	84
**HighMPCR-LowE**	**46.1** *(25.24)*	232	**39.66** *(18.87)*	76	**49.08** *(28.67)*	80	**49.39** *(26.04)*	76

Table S3. Average Treatment Effects of MPCR on individual contributions in Study 1: from OLS regression with robust standard errors in parentheses. *** p<0.005, ** p<0.05, * p<0.1. Self-reported motivations taken from the post-experimental questionnaire, measured in 5-likert-scale questions, with answers ranging from "I fully agree" … to … "I fully disagree". For the analysis, questions were coded as dummy variables, with individuals receiving a 1 for a given motivation if they answered the question with either "I fully agree" or "I agree", and 0 otherwise. Specifically, the variables were measured in the following question: "Think about your decision regarding allocations to the Group Account. Why did you decide to contribute as much as you did?" *Mistrust*: "I did not trust much the other group members. I did not expect them to contribute much." *Selfishness*: "To get the highest payoff for myself." *Social welfare*: "To get the highest payoffs for the whole group." *Shared responsibility*: "I felt the responsibility to contribute so I would not let my group members down." *Fairness / social norm*: "Because contributing is the right thing to do, irrespective of what the other group members do." *For the complete questionnaire, please see instructions in the OSF repository.*

Dep. Var: individual contribution (in % of endowment)	(1) Without individual controls	(2) With individual controls	(3) With individual controls & interactions
HighMPCR	-0.691	-0.686	-1.127
	(2.918)	(2.251)	(6.211)
HighMPCR-LowE	4.723*	2.683	2.151
	(2.853)	(2.314)	(5.905)
General Population	-3.647	-8.844***	-9.064***
	(2.837)	(2.337)	(2.393)
Students [online]	0.0839	-2.222	-2.173
	(3.039)	(2.357)	(2.381)
selfishness		-6.119***	-6.659*
		(2.120)	(3.703)
mistrust		-14.98***	-13.69***
		(2.073)	(3.443)
social welfare		24.49***	23.60***
		(2.158)	(3.824)
shared responsibility		5.635**	7.432**
		(2.190)	(3.559)
fairness / social norm		5.628**	3.520
		(2.328)	(3.789)
HighMPCR * selfishness			0.0183
			(5.112)
HighMPCR-LowE * selfishness			1.129
			(5.306)
HighMPCR * mistrust			-3.367
			(5.010)
HighMPCR-LowE * mistrust			0.483
			(5.133)
HighMPCR * social welfare			7.410
			(5.271)
HighMPCR-LowE * social welfare			-4.591
			(5.347)
HighMPCR * shared responsibility			-2.894
			(5.099)
HighMPCR-LowE * shared responsibility			-2.941
			(5.527)
HighMPCR * fairness / social norm			-0.369
			(5.365)
HighMPCR-LowE * fairness / social norm			7.052
			(5.870)
Constant	43.45***	38.35***	38.52***
	(2.688)	(3.073)	(4.408)
Observations	*716*	*716*	*716*
R-squared	*0.008*	*0.387*	*0.394*

Table S4. Average Treatment Effects of MPCR on individual expectations in Study 1: from OLS regression with robust standard errors in parentheses. *** p<0.005, ** p<0.05, * p<0.1. Self-reported motivations taken from the post-experimental questionnaire, measured in 5-likert-scale questions, with answers ranging from "I fully agree" … to … "I fully disagree". For the analysis, questions were coded as dummy variables, with individuals receiving a 1 for a given motivation if they answered the question with either "I fully agree" or "I agree", and 0 otherwise. Specifically, the variables were measured in the following question: "Think about your decision regarding allocations to the Group Account. Why did you decide to contribute as much as you did?" *Mistrust*: "I did not trust much the other group members. I did not expect them to contribute much." *Selfishness*: "To get the highest payoff for myself." *Social welfare*: "To get the highest payoffs for the whole group." *Shared responsibility*: "I felt the responsibility to contribute so I would not let my group members down." *Fairness / social norm*: "Because contributing is the right thing to do, irrespective of what the other group members do." *For the complete questionnaire, please see instructions in the OSF repository.*

Dep. Var: individual expectation (in % of endowment)	(1) Without individual controls	(2) With individual controls	(3) With individual controls & interactions
HighMPCR	-1.237	-0.642	0.556
	(2.269)	(1.978)	(5.144)
HighMPCR-LowE	1.598	0.994	6.977
	(2.217)	(2.041)	(5.307)
General Population	-5.149**	-7.245***	-7.302***
	(2.246)	(2.080)	(2.097)
Students [online]	0.693	-0.0182	0.161
	(2.373)	(2.098)	(2.103)
selfishness		-0.939	0.878
		(1.872)	(3.159)
mistrust		-14.87***	-12.69***
		(1.720)	(2.782)
social welfare		10.28***	10.95***
		(1.834)	(3.052)
shared responsibility		4.352**	6.913**
		(1.962)	(3.102)
fairness / social norm		1.139	-0.649
		(2.031)	(3.108)
HighMPCR * selfishness			-2.901
			(4.409)
HighMPCR-LowE * selfishness			-2.676
			(4.649)
HighMPCR * mistrust			-3.288
			(4.098)
HighMPCR-LowE * mistrust			-2.339
			(4.244)
HighMPCR * social welfare			6.616
			(4.337)
HighMPCR-LowE * social welfare			-8.403*
			(4.500)
HighMPCR * shared responsibility			-2.987
			(4.489)
HighMPCR-LowE * shared responsibility			-4.501
			(4.863)
HighMPCR * fairness / social norm			-0.382
			(4.578)
HighMPCR-LowE * fairness / social norm			5.738
			(5.024)
Constant	45.94***	46.35***	43.69***
	(2.124)	(2.597)	(3.672)
Observations	*716*	*716*	*716*
R-squared	*0.013*	*0.222*	*0.238*

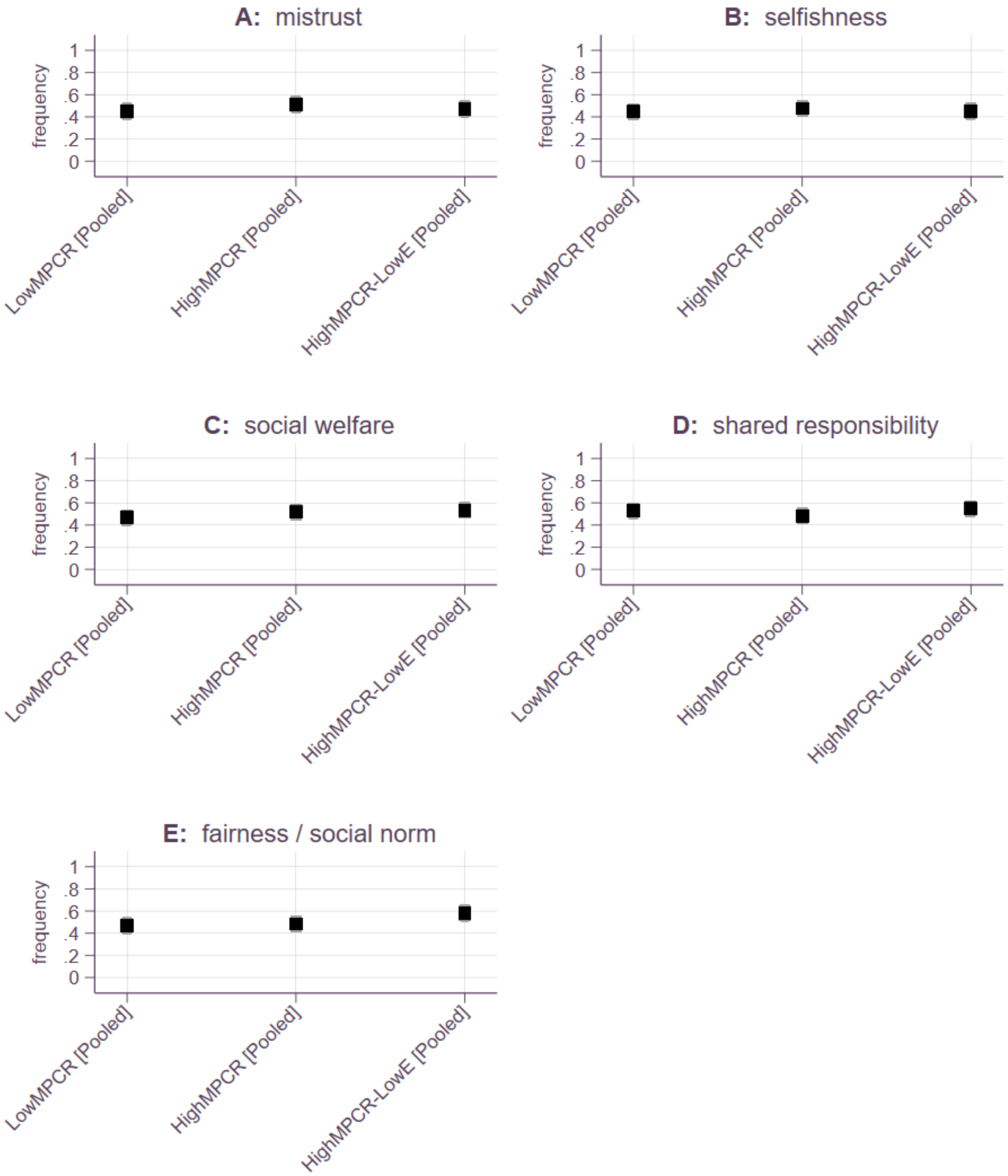

Figure S1. Frequency of self-reported motivations for each treatment in the pooled sample in Study 1. Self-reported motivations taken from the post-experimental questionnaire, measured in 5-likert-scale questions, with answers ranging from "I fully agree" … to … "I fully disagree". For the analysis, questions were coded as dummy variables, with individuals receiving a 1 for a given motivation if they answered the question with either "I fully agree" or "I agree", and 0 otherwise. Specifically, the variables were measured in the following question: "Think about your decision regarding allocations to the Group Account. Why did you decide to contribute as much as you did?" *Mistrust*: "I did not trust much the other group members. I did not expect them to contribute much." *Selfishness*: "To get the highest payoff for myself." *Social welfare*: "To get the highest payoffs for the whole group." *Shared responsibility*: "I felt the responsibility to contribute so I would not let my group members down." *Fairness / social norm*: "Because contributing is the right thing to do, irrespective of what the other group members do." *For the complete questionnaire, please see instructions in the OSF repository.*
Note: All p-values > 0.1 from two-sample t-tests for comparing the frequency of self-reported motivations between treatments, with the exception of the prevalence of fairness / social norm (Panel E), p-value = 0.01 for comparing *LowMPCR* to *HighMPCR-LowE*.

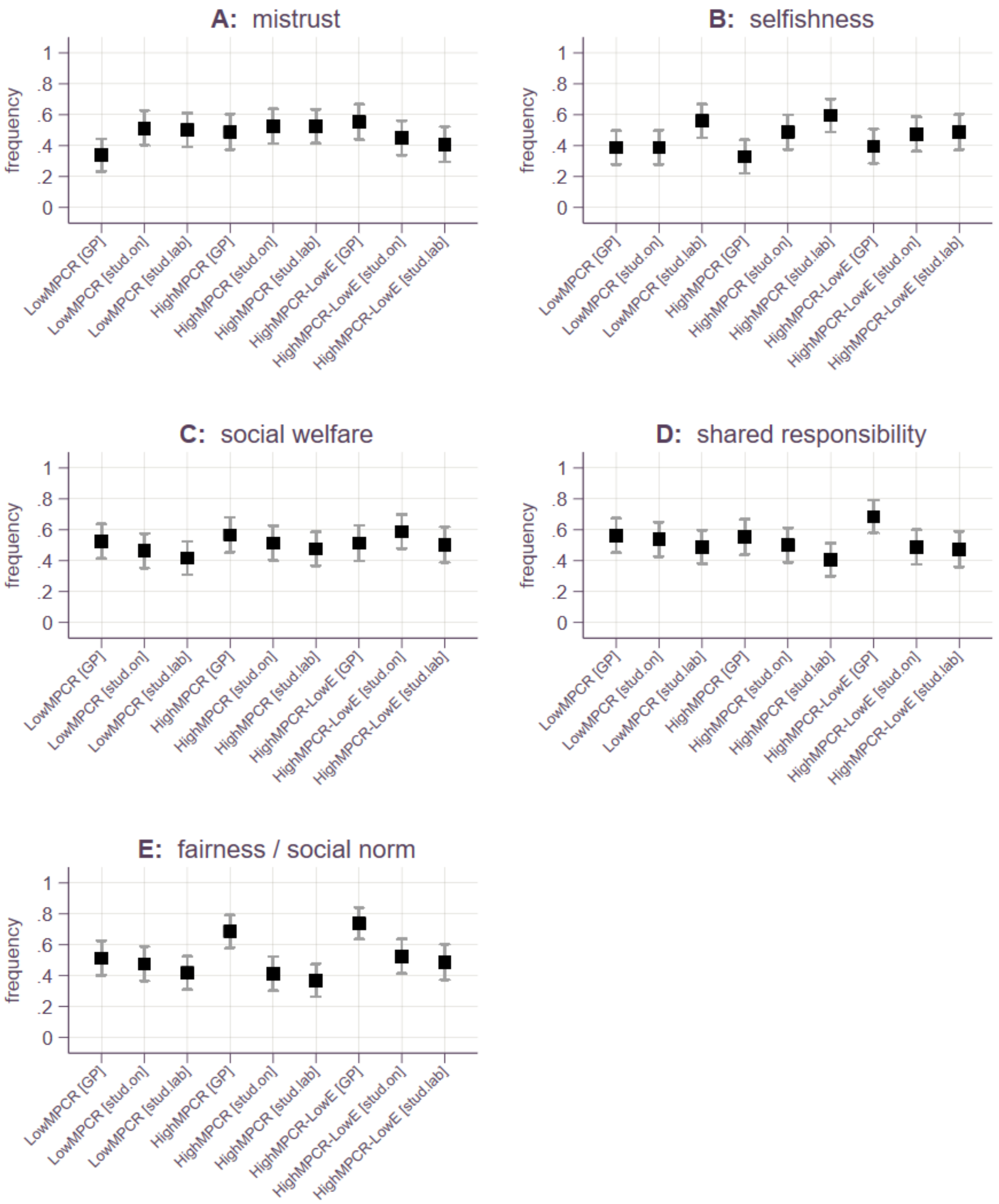

Figure S2. Frequency of self-reported motivations for each treatment and each sample separately in Study 1. Self-reported motivations taken from the post-experimental questionnaire, measured in 5-likert-scale questions, with answers ranging from "I fully agree" … to … "I fully disagree". For the analysis, questions were coded as dummy variables, with individuals receiving a 1 for a given motivation if they answered the question with either "I fully agree" or "I agree", and 0 otherwise. Specifically, the variables were measured in the following question: "Think about your decision regarding allocations to the Group Account. Why did you decide to contribute as much as you did?" *Mistrust*: "I did not trust much the other group members. I did not expect them to contribute much." *Selfishness*: "To get the highest payoff for myself." *Social welfare*: "To get the highest payoffs for the whole group." *Shared responsibility*: "I felt the responsibility to contribute so I would not let my group members down." *Fairness / social norm*: "Because contributing is the right thing to do, irrespective of what the other group members do." *For the complete questionnaire, please see instructions in the OSF repository.*

Table S5. Average Treatment Effects of MPCR on individual contributions in Study 1, for each sample separately: from OLS regression with robust standard errors in parentheses. *** p<0.005, ** p<0.05, * p<0.1

Dep. Var: individual contribution (in % of endowment)	(1) General Population	(2) Students [online]	(3) Students [lab]
HighMPCR	1.991	0.463	-4.226
	(4.767)	(5.156)	(5.209)
HighMPCR-LowE	2.741	8.475*	2.932
	(4.502)	(5.075)	(5.209)
Constant	39.57***	41.90***	45.23***
	(3.360)	(3.092)	(3.682)
Observations	*232*	*240*	*244*
R-squared	*0.002*	*0.013*	*0.008*

Table S6. Average Treatment Effects of MPCR on individual expectations in Study 1, for each sample separately: from OLS regression with robust standard errors in parentheses. *** p<0.005, ** p<0.05, * p<0.1

Dep. Var: individual expectation (in % of endowment)	(1) General Population	(2) Students [online]	(3) Students [lab]
HighMPCR	-0.974	3.700	-6.119
	(3.793)	(3.784)	(4.148)
HighMPCR-LowE	-2.342	5.325	1.847
	(3.328)	(3.910)	(4.186)
Constant	42***	43.75***	47.55***
	(2.528)	(2.239)	(2.935)
Observations	*232*	*240*	*244*
R-squared	*0.002*	*0.008*	*0.016*

Table S7. Correlations of expectations and contributions in Study 1: from OLS regression with robust standard errors in parentheses. *** p<0.005, ** p<0.05

	(1)	(2)	(3)	(4)
Dep. Var: individual contribution (in % of endowment)	Pooled Sample	General Population	Students [online]	Students [lab]
Expectation	0.868***	0.998***	0.803***	0.811***
	(0.0631)	(0.0530)	(0.143)	(0.106)
HighMPCR	-2.876	1.566	-11.25	-1.176
	(3.863)	(3.478)	(8.384)	(7.139)
HighMPCR-LowE	7.186	4.582	3.413	10.59
	(4.364)	(4.041)	(8.730)	(8.739)
Expectation**HighMPCR*	0.0750	0.0340	0.184	0.0462
	(0.0828)	(0.0708)	(0.171)	(0.149)
Expectation**HighMPCR-LowE*	-0.0837	0.0125	0.0161	-0.185
	(0.0965)	(0.0979)	(0.178)	(0.179)
Constant	3.691	-2.329	6.783	6.647
	(2.906)	(2.433)	(7.239)	(4.813)
Observations	*716*	*232*	*240*	*244*
R-squared	*0.468*	*0.619*	*0.456*	*0.393*

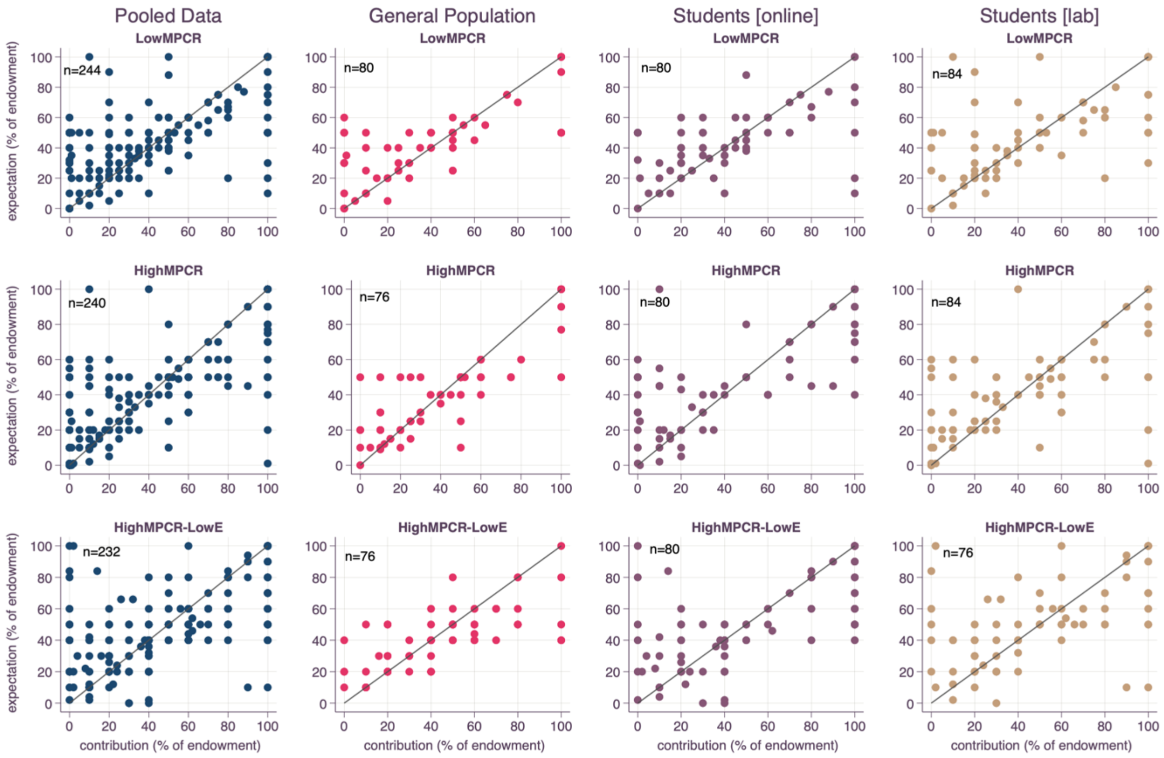

Figure S3. Scatter plot showing the relationship between individual contributions and individual expectations: for each treatment and each sample of Study 1 separately.

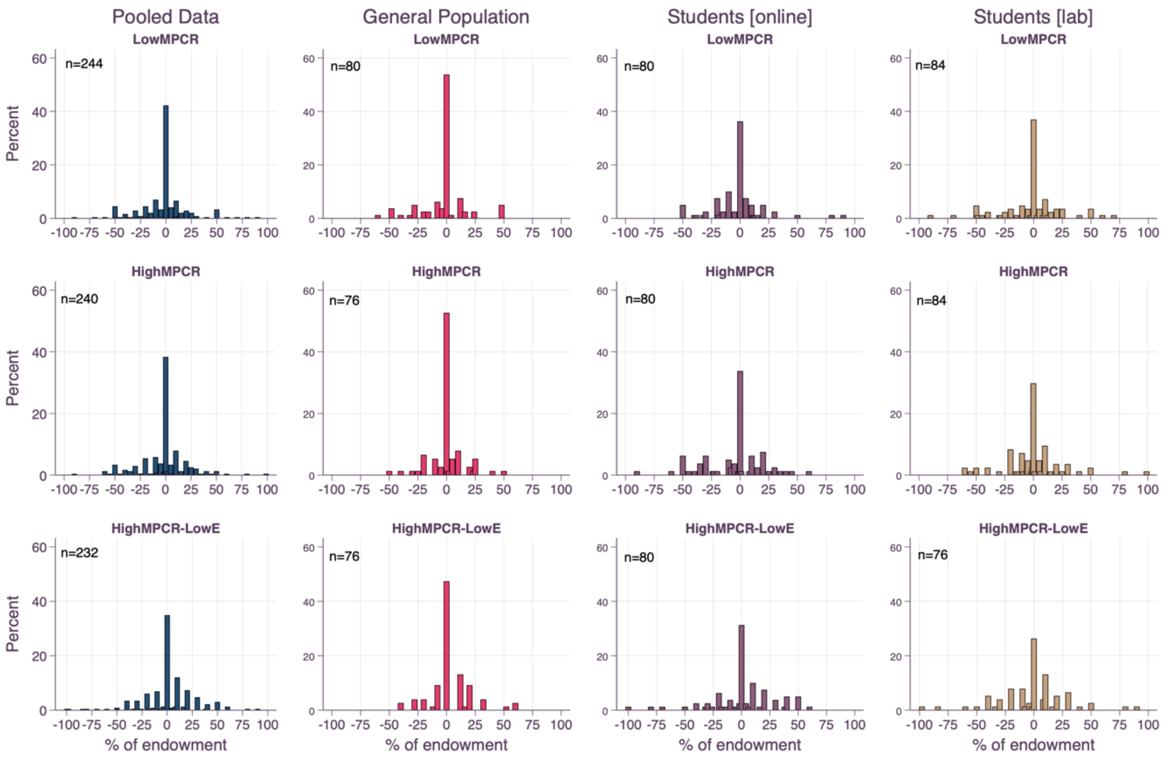

Figure S4. Distributions of difference between individual expectation of behavior of others from individual contribution. In percent of endowment, for each treatment and each sample of Study 1 separately, calculated as deviation = contribution – expectation. Positive values indicate one's own contribution is higher than the average expectation of others, negative values indicate one's own contribution is lower than the average expectation of others.

3.1. Tests for equality of distributions in Study 1

We present here the exact p-values from Kolmogorov-Smirnov tests for equality of distributions, comparing across treatments (across MPCR levels) the distributions for individual contributions, the distributions of individual expectations and the distributions of deviations of individual expectations from individual contributions.

Tests and p-values to Figure 5.

- Comparisons for distributions of individual contributions:

Pooled Sample: p-values for comparison of *LowMPCR* with *HighMPCR* and with *HighMPCR-LowE* respectively are 0.51 and 0.28. ***General Population***: p-values for comparison of *LowMPCR* with *HighMPCR* and with *HighMPCR-LowE* respectively are 0.95 and 0.65. ***Students [lab]***: p-values for comparison of *LowMPCR* with *HighMPCR* and with *HighMPCR-LowE* respectively are 0.93 and 0.79. ***Students [online]***: p-values for comparison of *LowMPCR* with *HighMPCR* and with *HighMPCR-LowE* respectively are 0.17 and 0.054.

- Comparison of distributions of individual expectations:

Pooled Sample: p-values for comparison of *LowMPCR* with *HighMPCR* and with *HighMPCR-LowE* respectively are 0.19 and 0.47. ***General Population***: p-values for comparison of *Low MPCR* with *HighMPCR* and with *HighMPCR-LowE* respectively are 0.81 and 0.41. ***Students [lab]***: p-values for comparison of *LowMPCR* with *HighMPCR* and with *HighMPCR-LowE)* respectively are 0.59 and 0.96. ***Students [online]***: p-values for comparison of *LowMPCR* with *HighMPCR* and with *HighMPCR-LowE* respectively are 0.24 and 0.12.

Tests and p-values to Figure S5.

- Comparisons in the distributions for deviations of individual expectations from individual contributions.

Pooled Sample: p-values for comparison of *LowMPCR* with *HighMPCR* and with *HighMPCR-LowE* respectively are 1.0 and 0.03. ***General Population***: p-values for comparison of *LowMPCR* with *HighMPCR* and with *HighMPCR-LowE)* respectively are 0.92 and 0.42. ***Students [lab]***: p-values for comparison of *LowMPCR* with *HighMPCR* and with *HighMPCR-LowE* respectively are 0.98 and 0.76. ***Students [online]***: p-values for comparison of *LowMPCR* with *HighMPCR* and with *HighMPCR-LowE)* respectively are 0.56 and 0.33.

3.2. Results and discussion of check of assumptions to use linear regression models (OLS):

We present here the various tests of model assumptions based on the OLS model fitted for Figure 3 in the main text, and Table S1 model (1) presented in section 3 of this document.

Homogeneity of variance: We run a Breusch-Pegan / Cook-Weisberg test for heteroskedasticity with the null hypothesis of constant (equal) variance: $\chi^2(1) = 1.51, p-value = 0.22$. Thus, we cannot reject the null hypothesis of homoscedasticity. Nevertheless, all models use robust standard errors (to account for any possible violation of uniformity of variance).

Normality of residuals: We present a standardized normal probability plot (p-p plot) in Fig. S1. The figure indicates (small) deviations from normality. Given our large sample size (n=716), however, and large degrees of freedom (df=711), it is reasonable to assume that asymptotic theory (Central Limit Theorem) will hold, such that regression coefficients on average are normally distributed. Both Knief & Forstmeier (53) and Lumley et al. (54) demonstrate using simulations that both t-tests and linear regressions are valid methods to derive inference even in situations where the normality assumption is violated (i.e. p-values remaining reliable and models performing well in terms of power). Therefore, here and throughout all other regression analysis in Studies 1 and 2, we rely on OLS regressions.

For robustness, we also provide here non-parametric tests that do no assume an underlying distribution of the data. Specifically, we report here the results from two-sample ranksum (Mann-Whitney) tests (comparing *LowMPCR* vs *HighMPCR* and *LowMPCR* vs *HighMPCR-LowE*, respectively). P-values for the Pooled sample are 0.59 and 0.14. P-values for the General Population sample 0.62 and 0.45. P-values for the student online are 0.39 and 0.50. P-values for the student laboratory sample are 0.58 and 0.29. In summary, none of these differences are significant at a conventional p-value < 0.05, and all results are qualitatively robust to both the OLS regressions and two-sample t-test comparisons.

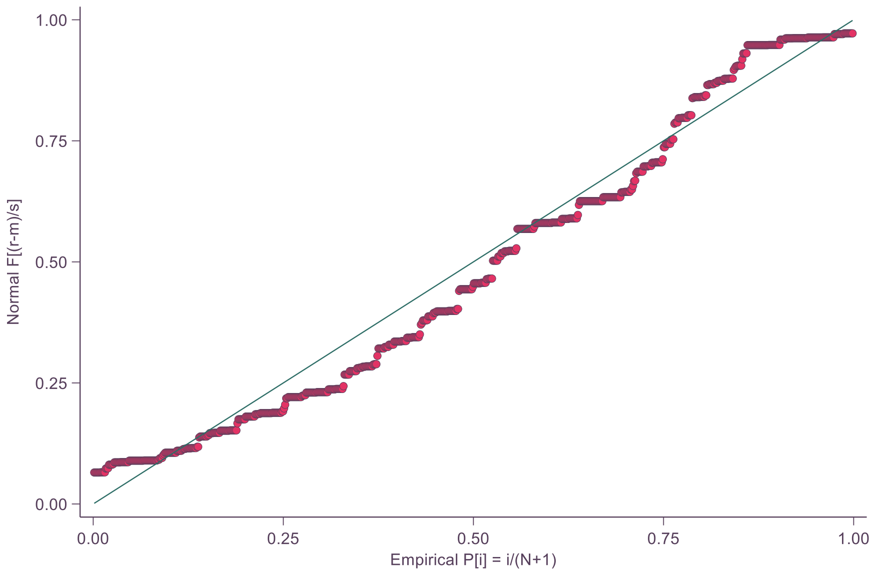

Figure S5. Standardized normal probability plot of model residuals

4. Study 2 - Additional Analysis

Table S8. Contributions in Study 2: Summary statistics of average individual contributions in Study 2, in % of endowments, by treatment and sample. Std. deviations in parentheses. For comparison of contributions in *LowMPCR* with *HighMPCR* we report p-values from two-sample t-tests for each treatment condition separately. P-value for *EXO(passive)* is 0.72. P-value for *EXO(active)* is 0.55. And p-value for *EndoMPCR* is 0.52. In summary, none of these differences are significant at a conventional p-value < 0.05.

	**EXO(passive)**	*n*	**EXO(active)**	*n*	**EndoMPCR**	*n*
**LowMPCR**	**38.88** *(26.94)*	80	**38.25** *(27.00)*	84	**39.62** *(28.84)*	72
**HighMPCR**	**40.51** *(29.04)*	76	**35.48** *(32.35)*	80	**42.09** *(28.66)*	248

Table S9. Expectations in Study 2: Summary statistics of average individual expectations in Study 2: in percentage of endowments, by treatment and sample. Std. deviations in parentheses. For the comparison of expectation in *LowMPCR* with *HighMPCR* of the *EXO(passive)* treatment, the p-value from a two-sample t-test is 0.84.

	**EXO(passive)**	*n*
**LowMPCR**	**41.01** *(24.85)*	80
**HighMPCR**	**41.83** *(24.16)*	76

Table S10. Average Treatment Effects of MPCR on individual contributions in Study 2: from OLS regression with robust standard errors in parentheses. *** p<0.005, ** p<0.05, * p<0.1. Self-reported motivations taken from the post-experimental questionnaire, measured in 5-likert-scale questions, with answers ranging from “I fully agree” … to … “I fully disagree”. For the analysis, questions were coded as dummy variables, with individuals receiving a 1 for a given motivation if they answered the question with either “I fully agree” or “I agree”, and 0 otherwise. Specifically, the variables were measured in the following question: “Think about your decision regarding allocations to the Group Account. Why did you decide to contribute as much as you did?” *Mistrust*: “I did not trust much the Type 2 group members. I did not expect them to contribute much.” *Selfishness*: “To get the highest payoff for myself.” *Social welfare*: “To get the highest payoffs for the whole group, both for Type 1 and Type 2.” *Shared responsibility*: “I felt the responsibility to contribute so I would not let my group members down.” *Fairness / social norm*: “Because contributing is the right thing to do, irrespective of what the Type 1 and Type 2 group members do.” Notice that in the experiment we refer to providers as Type 2, and to beneficiaries as Type 1. *For the complete questionnaire, please see instructions in the OSF repository.*

	EXO(passive)			EXO(active)			EndoMPCR		
	(1)	(2)	(3)	(4)	(5)	(6)	(7)	(8)	(9)
Dep. Var: individual contribution (in % of endowment)	Without controls	With controls	With controls & interactions	Without controls	With controls	With controls & interactions	Without controls	With controls	With controls & interactions
HighMPCR	1.638<br>(4.491)	-2.138<br>(4.032)	-5.402<br>(9.825)	-2.775<br>(4.664)	-2.133<br>(4.241)	-2.731<br>(10.01)	2.468<br>(3.844)	2.235<br>(3.368)	16.11**<br>(7.443)
mistrust		-16.24***<br>(4.292)	-20.00***<br>(5.853)		-11.15**<br>(4.438)	-9.694<br>(5.876)		-<br>10.88***<br>(3.101)	-14.30**<br>(6.394)
selfishness		-9.268*<br>(5.068)	-5.400<br>(6.970)		-9.132*<br>(4.847)	-7.119<br>(5.566)		-8.018**<br>(3.464)	1.582<br>(7.214)
social welfare		12.95***<br>(4.081)	10.68*<br>(5.647)		10.22**<br>(4.284)	2.457<br>(5.473)		15.14***<br>(3.011)	24.12***<br>(5.925)
shared responsibility		0.824<br>(5.025)	7.272<br>(7.277)		0.637<br>(6.314)	4.648<br>(7.920)		-9.563**<br>(4.045)	-10.15<br>(8.779)
fairness / social norm		5.115<br>(4.843)	-0.355<br>(6.685)		12.90**<br>(5.902)	12.02<br>(7.625)		10.56***<br>(3.650)	16.63**<br>(7.447)
HighMPCR * mistrust			7.050<br>(8.594)			-1.625<br>(9.037)			4.028<br>(7.298)
HighMPCR * selfishness			-6.325<br>(10.27)			-5.187<br>(10.02)			-12.29<br>(8.180)
HighMPCR * social welfare			5.298<br>(8.311)			16.80**<br>(8.493)			-11.77*<br>(6.875)
HighMPCR * shared responsibility			-9.800<br>(9.969)			-9.452<br>(12.28)			0.703<br>(9.871)
HighMPCR * fairness / social norm			8.785<br>(9.678)			1.009<br>(11.49)			-7.751<br>(8.513)
Constant	38.88***<br>(3.013)	41.77***<br>(4.911)	42.26***<br>(6.628)	38.25***<br>(2.946)	33.05***<br>(5.468)	33.13***<br>(5.751)	39.62***<br>(3.385)	37.86***<br>(3.897)	27.08***<br>(6.373)
Observations	*156*	*156*	*156*	*164*	*164*	*164*	*320*	*320*	*320*
R-squared	*0.001*	*0.224*	*0.236*	*0.002*	*0.234*	*0.255*	*0.001*	*0.175*	*0.192*

Table S11. Average Treatment Effects of MPCR on individual expectations in Study 2: from OLS regression with robust standard errors in parentheses. *** p<0.005, ** p<0.05, * p<0.1. Self-reported motivations taken from the post-experimental questionnaire, measured in 5-likert-scale questions, with answers ranging from “I fully agree” … to … “I fully disagree”. For the analysis, questions were coded as dummy variables, with individuals receiving a 1 for a given motivation if they answered the question with either “I fully agree” or “I agree”, and 0 otherwise. Specifically, the variables were measured in the following question: “Think about your decision regarding allocations to the Group Account. Why did you decide to contribute as much as you did?” *Mistrust*: “I did not trust much the Type 2 group members. I did not expect them to contribute much.” *Selfishness*: “To get the highest payoff for myself.” *Social welfare*: “To get the highest payoffs for the whole group, both for Type 1 and Type 2.” *Shared responsibility*: “I felt the responsibility to contribute so I would not let my group members down.” *Fairness / social norm*: “Because contributing is the right thing to do, irrespective of what the Type 1 and Type 2 group members do.” Notice that in the experiment we refer to providers as Type 2, and to beneficiaries as Type 1. *For the complete questionnaire, please see instructions in the OSF repository.*

	EXO(passive)		
Dep. Var: individual expectation (in % of endowment)	(1) Without controls	(2) With controls	(3) With controls & interactions
HighMPCR	0.816	-1.733	-3.076
	(3.925)	(3.698)	(8.218)
mistrust		-12.92***	-16.82***
		(3.789)	(5.433)
selfishness		-0.0244	2.626
		(4.509)	(6.574)
social welfare		12.55***	14.16**
		(3.774)	(5.438)
shared responsibility		2.200	6.321
		(4.601)	(7.434)
fairness / social norm		3.812	-0.574
		(4.461)	(6.587)
HighMPCR * mistrust			6.946
			(7.601)
HighMPCR * selfishness			-3.835
			(9.432)
HighMPCR * social welfare			-3.076
			(7.604)
HighMPCR * shared responsibility			-5.742
			(9.548)
HighMPCR * fairness / social norm			7.486
			(9.011)
Constant	41.01***	38.73***	38.91***
	(2.779)	(4.318)	(6.347)
Observations	*156*	*156*	*156*
R-squared	*0.000*	*0.174*	*0.185*

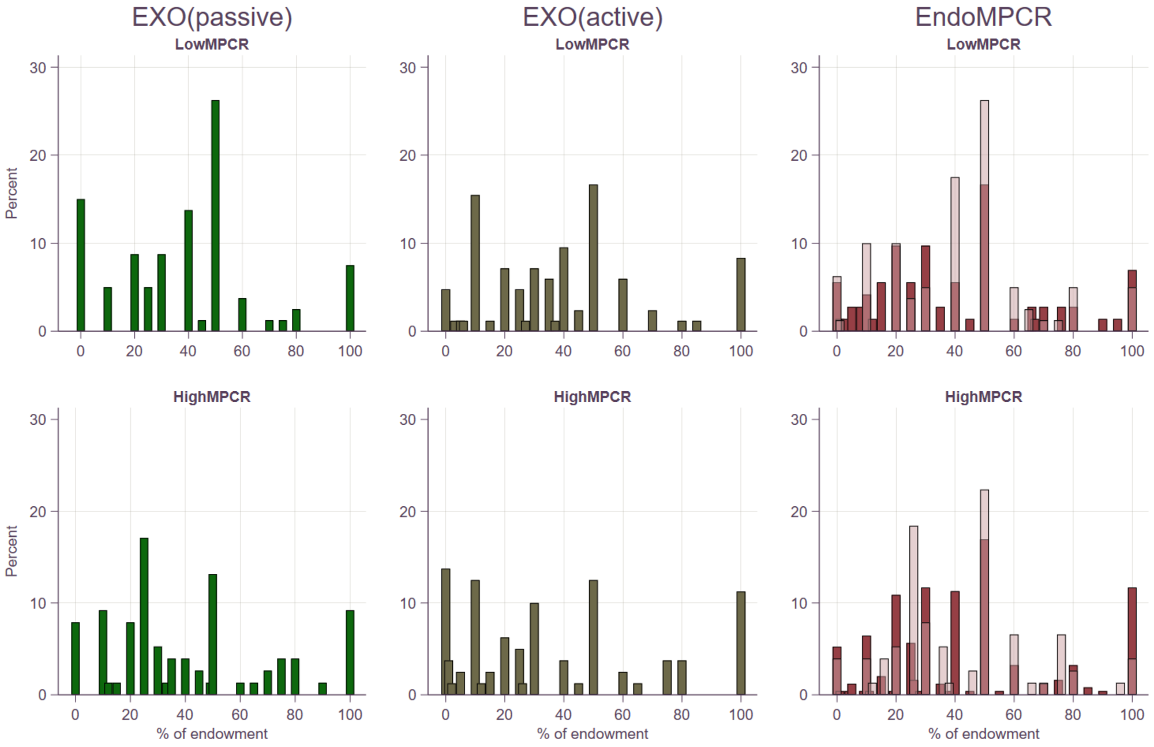

Figure S6. Distributions of individual contributions by providers in percent of endowment in Study 2. EXO(passive) - individual contributions (dark-shaded), expectations of behavior of other providers (light-shaded). Note: We report Kolmogorov-Smirnov tests for equality of distributions of individual contributions. P-value for comparison of *EXO(passive)-LowMPCR* with *EXO(passive)-HighMPCR* is 0.55; p-value for comparison of *EXO(active)-LowMPCR* with *EXO(active)-HighMPCR* is 0.33; and p-value for comparison of *EndoMPCR-LowMPCR* with *EndoMPCR-HighMPCR* is 0.57.

Table S12. Correlations of expectations and contributions in Study 2: Data from OLS regression with robust standard errors at the individual level. ** p<0.005, * p<0.05

	(1)
Dep. Var: individual contribution (in % of endowment)	EXO(passive)
Expectation	0.872***
	(0.0832)
HighMPCR	0.398
	(4.321)
Expectation * HighMPCR	0.0126
	(0.116)
Constant	3.106
	(3.380)
Observations	*156*
R-squared	*0.592*

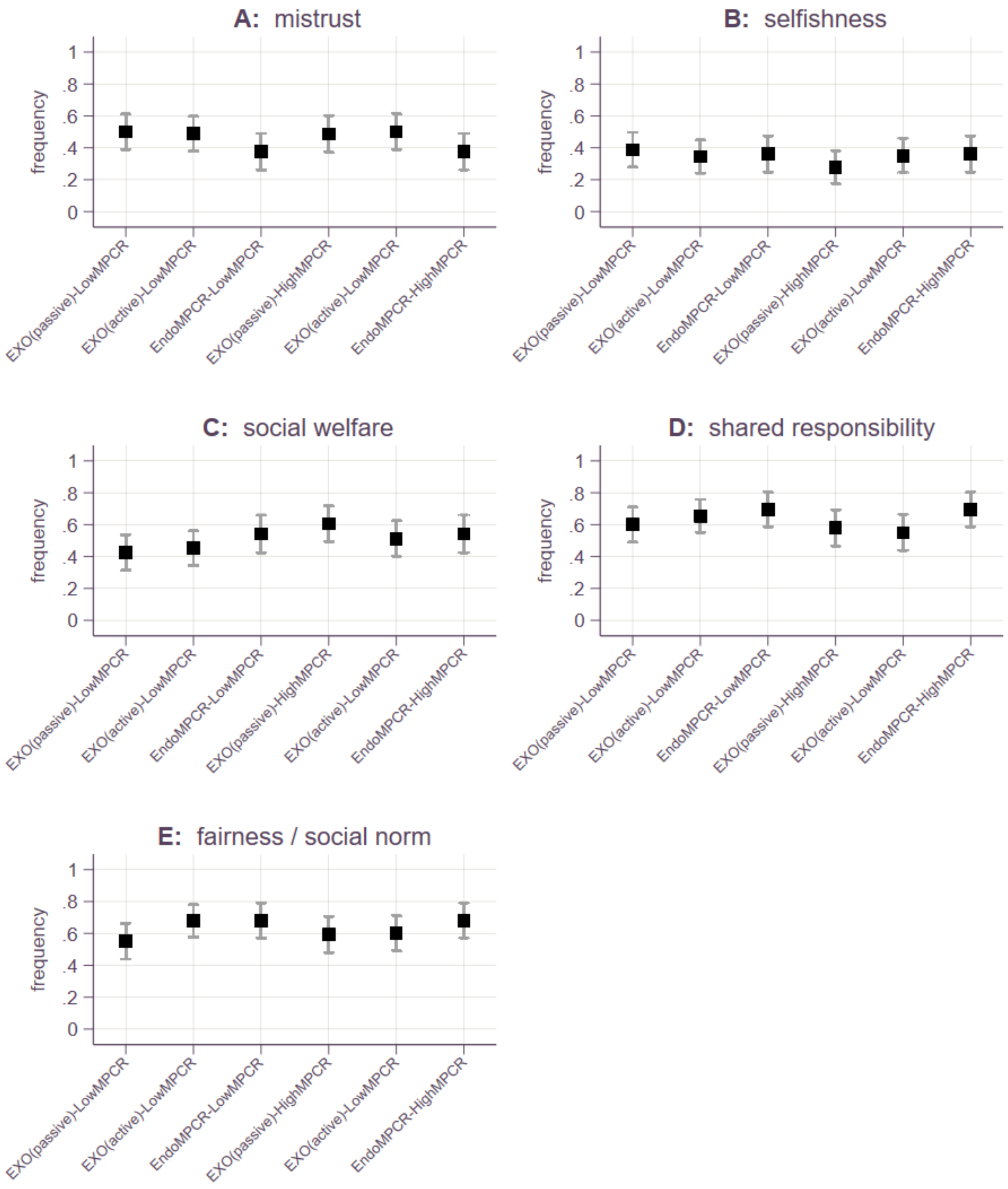

Figure S7. Frequency of self-reported motivations for each treatment in Study 2. Self-reported motivations taken from the post-experimental questionnaire, measured in 5-likert-scale questions, with answers ranging from "I fully agree" … to … "I fully disagree". For the analysis, questions were coded as dummy variables, with individuals receiving a 1 for a given motivation if they answered the question with either "I fully agree" or "I agree", and 0 otherwise. Specifically, the variables were measured in the following question: "Think about your decision regarding allocations to the Group Account. Why did you decide to contribute as much as you did?" *Mistrust*: "I did not trust much the Type 2 group members. I did not expect them to contribute much." *Selfishness*: "To get the highest payoff for myself." *Social welfare*: "To get the highest payoffs for the whole group, both for Type 1 and Type 2." *Shared responsibility*: "I felt the responsibility to contribute so I would not let my group members down." *Fairness / social norm*: "Because contributing is the right thing to do, irrespective of what the Type 1 and Type 2 group members do." Notice that in the experiment we refer to providers as Type 2, and to beneficiaries as Type 1. *For the complete questionnaire, please see instructions in the OSF repository.*

4.1. Inattention, randomness, or confusion in Study 2?

In total, 8.9 percent of participants (57 of 640) report not having fully understood the decision task. This number is slightly higher than in Study 1, which is to be expected given the more complex group-to-group experimental task and thus more complex instructions. All results are qualitatively robust to dropping these participants from the analyses.

As in Study 1, we test whether contributions by providers follow a uniform distribution, what one might expect if choices were purely random. All p-values < 0.0001 from one-sided Kolmogorov-Smirnov tests against uniformly distributed random integer variates on the interval [0,100]).

5. Robustness Analysis of Treatments with reversed order of soliciting contribution and expectation decisions

Table S13. Summary statistics of average individual contributions in the GP and GP *robustness* data collections: in percentage of endowments. Std. deviations in parentheses. All reported p-values are from two-sided t-tests. **Horizontal comparisons:** P-values for comparisons between the robustness and the original samples are 0.092 for *LowMPCR*, 0.33 for *HighMPCR* and 0.71 for *HighMPCR-LowE,* respectively. **Vertical comparison:** P-values for between treatment comparisons in the robustness sample of *LowMPCR* with *HighMPCR* and respectively with *HighMPCR-LowE* are 0.31 and 0.018, respectively.

	General Population (robustness) – contributions first	*n*	***General Population – expectations first***	*n*
LowMPCR	**32** *(26.25)*	80	**39.58** *(30.05)*	80
HighMPCR	**36.71** *(31.47)*	76	**41.57** *(29.48)*	76
HighMPCR-LowE	**44.12** *(37.04)*	80	**42.32** *(26.12)*	76

Table S14. Summary statistics of average individual expectations in the GP and GP *Robustness* data collections: in % of endowments. Std. deviations in parentheses. **Horizontal comparisons:** The difference between the *LowMPCR [GP rob]* and the *LowMPCR [GP]* is 11.78%-points (p-value is 0.0003; all reported p-values are from two-sided t-tests). There is a 5.29%-points difference between *HighMPCR [GP rob]* and *HighMPCR [GP]* (p-value is 0.16) and a 3.99%-points difference between *HighMPCR-LowE [GP rob]* and *HighMPCR-LowE [GP]* (p-value is 0.31). **Vertical comparisons:** For the GP robustness treatments there is a 5.52%-points increase in expectations moving from *Low MPCR* to *HighMPCR* (p-value is 0.08), and a 13.44%-points increase moving to *HighMPCR-LowE* (p-value is 0.0005). There is a 7.91%-points difference between *HighMPCR* and *HighMPCR-LowE* (p-value is 0.056).

	General Population (robustness) – contributions first	*n*	***General Population – expectations first***	*n*
LowMPCR	**30.21** *(17.48)*	80	**42** *(22.61)*	80
HighMPCR	**35.74** *(21.50)*	76	**41.03** *(24.65)*	76
HighMPCR-LowE	**43.65** *(29.00)*	80	**39.66** *(18.87)*	76

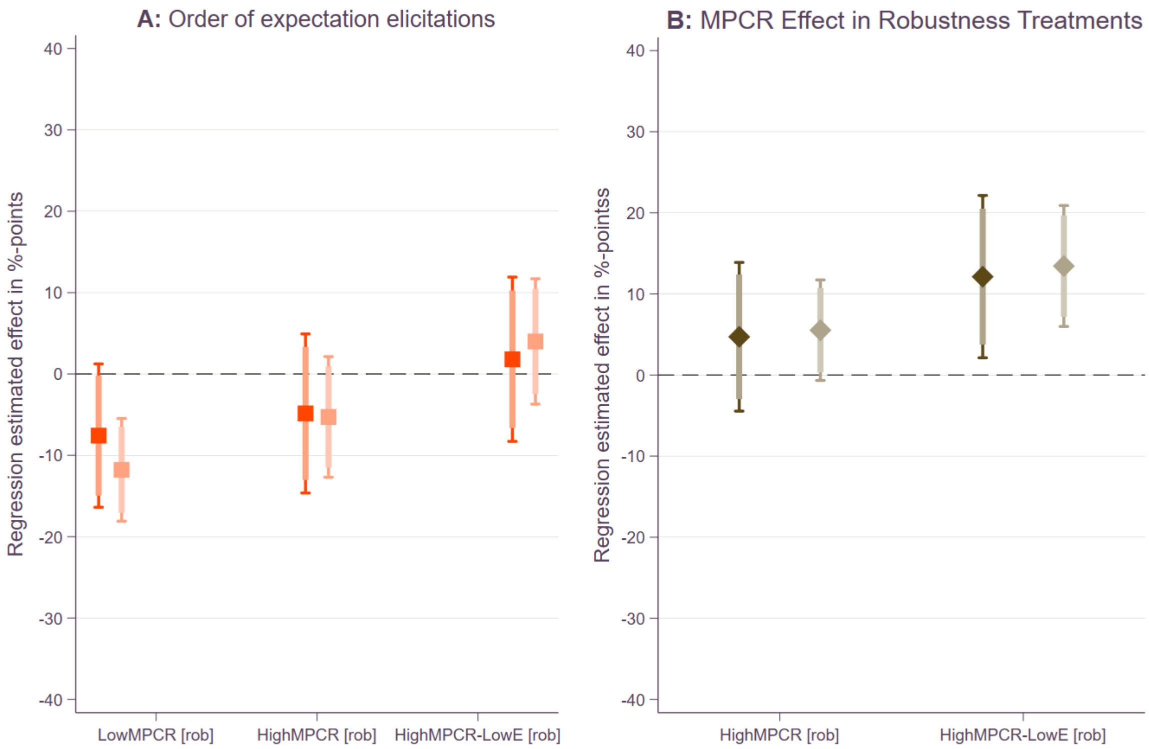

Figure S8. Point estimates and confidence intervals (90% CI indicated by shaded spikes, and 95% CI indicated by the "caps" at the ends of each side of the confidence intervals) from OLS regression with robust standard errors for the behavioral outcomes: contribution to the public good in percentage of endowment (dark-shaded) and individual expectations of the behavior of others (light-shaded). **Panel A:** Treatment effects from changing order of eliciting contributions and expectations. Models are estimated separately for comparing each of the treatments of the General Population *robustness* treatments (with contribution decisions first) to its respective counterpart with expectations first. **Panel B:** Treatment effects from changing MPCR levels in the General Population *robustness* sample. Explanatory variables are the treatment dummies (*HighMPCR [rob]* and *HighMPCR-LowE [rob])* indicating the effect of the respective treatment condition as compared to the *LowMPCR [rob]* condition. See Supporting Information Tables S15 and S16 for the full regression outputs behind this figure.

Table S15. Average Treatment Effects on individual contributions in General Population robustness data: from OLS regression with robust standard errors in parentheses. *** p<0.005, ** p<0.05, * p<0.1

	Timing of decision-making effect			MPCR effect
Dep. Var: individual contributions (in % of endowment)	(1) Contributions first LowMPCR	(2) Contributions first HighMPCR	(3) Contributions first HighMPCR-LowE	(4) General Population *robustness*
LowMPCR [rob]	-7.575* (4.461)			
HighMPCR [rob]		-4.855 (4.946)		4.711 (4.652)
HighMPCR-LowE [rob]			1.809 (5.111)	12.12** (5.076)
Constant	39.58*** (3.360)	41.57*** (3.381)	42.32*** (2.996)	32*** (2.936)
Reference category	*LowMPCR*	*HighMPCR*	*HighMPCR-LowE*	*LowMPCR [rob]*
Observations	*160*	*152*	*156*	*236*
R-squared	*0.018*	*0.006*	*0.001*	*0.025*

Table S16. Average Treatment Effects on individual expectations in General Population robustness data: from OLS regression with robust standard errors in parentheses. *** p<0.005, ** p<0.05, * p<0.1

	Timing of decision-making effect			MPCR effect
Dep. Var: individual expectation (in % of endowment)	(1) Contributions first LowMPCR	(2) Contributions first HighMPCR	(3) Contributions first HighMPCR-lowE	(4) General Population *robustness*
LowMPCR [rob]	-11.79***			
	(3.195)			
HighMPCR [rob]		-5.289		5.524*
		(3.753)		(3.147)
HighMPCR-LowE [rob]			3.992	13.44***
			(3.899)	(3.786)
Constant	42***	41.03***	39.66***	30.21***
	(2.528)	(2.828)	(2.164)	(1.955)
Reference category	*LowMPCR*	*HighMPCR*	*HighMPCR-LowE*	*LowMPCR [rob]*
Observations	*160*	*152*	*156*	*236*
R-squared	*0.079*	*0.013*	*0.007*	*0.055*

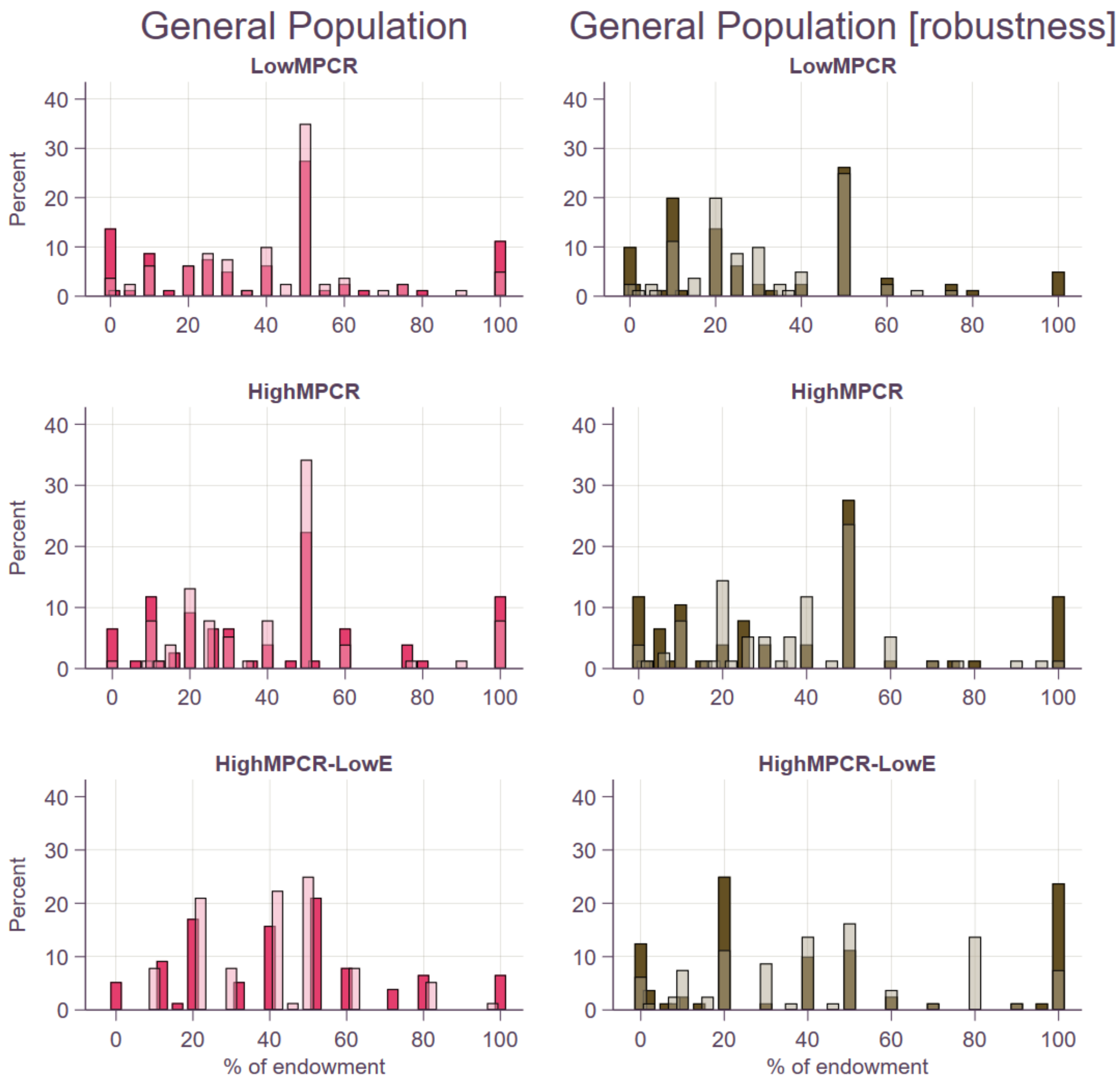

Figure S9. Distributions of individual contributions in Study 1: in percent of endowment (bold colors) and individual expectations of behavior of others in percent of endowment (light-shaded colors), for each treatment in the General Population separately. Kolmogorov-Smirnov tests for equality of distributions for individual contributions suggest that none of the comparisons in the distribution of contributions are significant at a p-value < 0.05. K-S tests for equality of distributions for individual expectations suggest only the comparisons between *LowMPCR* with *LowMPCR [rob]* (horizontal comparison) and between *LowMPCR [rob]* and *HighMPCR-LowE) [rob]* (vertical comparisons) are significant at a p-value < 0.05. Note that paired t-tests show that within a treatment, on average, individual contributions are not significantly different from individual expectations (all p-values from paired t-tests > 0.05).

Summary of results from robustness treatments:

(i) **Within the General population sample, average contributions are not systematically affected from whether expectations are elicited before or after contribution decisions.** Further, average expectations only differ in the Low MPCR environment.

(ii) **There is no systematic and consistent evidence that a higher MPCR with contributions first significantly increases public good provision:** The treatment *HighMPCR [rob]* does not result in significantly higher contributions or expectations as compared to *LowMPCR [rob]*. The difference in contributions and expectations between *HighMPCR-LowE [rob]* and *LowMPCR [rob]* is significant at p-value < 0.05, however, contributions in *HighMPCR-LowE [rev]* and *HighMPCR [rev]* are not significantly different from each other (p-value = 0.19). Further, this significant coefficient is not stable to multiple hypothesis correction (see Table S9 in section 6).

(iii) **No differences in the distribution of contributions between treatments:** The distributions of contributions are not significantly different comparing the different MPCR treatments with the reversed order; as well as comparing for each MPCR treatment the different order of decision makings (expectations first vs contributions first).

(iv) **Some, but no systematic differences in expectations about the average behavior of others**: average expectations as well as the distribution of expectation in the *LowMPCR [RevOrd]* treatment are significantly different from its counterpart *Low PCR* in Prolific and from *HighMPCR-LowE [RevOrd]*. All other pair-wise comparisons of expectations and distributions of expectations are insignificant.

In sum, there are no significant differences in contribution levels when comparing the treatments with expectations elicited first and the ones with contributions made first. Within the robustness experiments, all adjusted p-values show that no comparison is significantly different at p-value < 0.05 (correcting for multiple hypothesis testing, given the now large number of simultaneous hypothesis tests in study 1). We conclude from this additional analysis that the order in which participants are confronted with expectations and contribution decisions does not systematically affect cooperation levels nor treatment effects in our decision setting.

6. Multiple hypothesis correction

Since we are interested in the differences in contributions between the three low and high MPCR treatments for the pooled data, and each sample separately, this gives ten main comparisons that we are testing simultaneously in Study 1 (only considering average contributions). This relatively large number of simultaneous hypotheses tests increases the probability of false rejections of the null hypothesis and justifies correcting our analysis for the multiplicity of tested null hypotheses. Table S9 presents results for the p-value adjustments due to multiple hypothesis testing.

In column II we give for each sample and each MPCR comparison the difference in contributions in percentage of endowments. Column III shows the unadjusted (original) p-values. The last three columns show the adjusted p-values using different testing procedures for multiple hypothesis testing. The first is the procedure introduced in List et al. (55), which asymptotically controls the familywise error rate (which gives the probability of one or more false rejections when testing multiple null hypotheses). We additionally report p-values adjusted according to the classical Bonferroni and Holm corrections.

Table S17. Correction for multiple hypothesis testing in Study 1.

				Adjusted p-values		
		Difference (in % of endowment)	***Unadjusted p-value***	***List et al (2019)***	***Bonf***	***Holm***
Pooled	*LowMPCR v. HighMPCR*	-0.65	0.83	0.83	1	0.83
	LowMPCR v. HighMPCR-LowE	4.73	.099	0.18	0.4	0.2
GP	*LowMPCR v. HighMPCR*	1.99	0.68	0.90	1	1
	LowMPCR v. HighMPCR-LowE	2.74	0.54	0.95	1	1
**Students [online]**	*LowMPCR v. HighMPCR*	0.46	0.93	0.93	1	0.92
	LowMPCR v. HighMPCR-LowE	8.48	0.098	0.49	0.78	0.69
**Students [lab]**	*LowMPCR v. HighMPCR*	- 4.23	0.42	0.93	1	1
	LowMPCR v. HighMPCR-LowE	2.93	0.58	0.93	1	1
GP (RevOrd)	*LowMPCR v. HighMPCR*	4.71	0.31	0.87	1	1
	LowMPCR v. HighMPCR-LowE	12.13	0.02	0.11	0.12	0.12

7. Post-experimental questionnaire (survey questions)

Study 1:

1. How comprehensible did you find the instructions to this experiment (very ... to... not at all)?

2. Did you contribute to any charity in the last 12 months? (yes/no)

   If yes, what is your estimate of how much in total to all charities (approx.)?

3. Have you worked for any charity in the last 12 months?

   If yes, how many hours in total for all charities (approx.)?

4. Think about your decisions on contributions to the group account. Why did you decide to contribute as much as you did? Please indicate to what degree you agree with the following statements:

**I contributed as much as I did, because …**	**Strongly agree**	**Agree**	**Neutral**	**Disagree**	**Strongly disagree**
I followed the example in the instructions. Participants that contributed less than others received higher payoffs.					
I did not trust much the other group members. I did not expect them to contribute much.					
To get the highest payoff for myself.					
To get the highest payoff for the whole group.					
I wanted to contribute some but also save some.					
I felt the responsibility to contribute so I would not let my group members down.					
Because contributing is the right thing to do, irrespective of what other group members do.					
I did not understand the decision task.					

Study 2:

1. How comprehensible did you find the instructions to this experiment (very ... to... not at all)?

2. Did you contribute to any charity in the last 12 months? (yes/no)

   If yes, what is your estimate of how much in total to all charities (approx.)?

3. Have you worked for any charity in the last 12 months?

    If yes, how many hours in total for all charities (approx.)?

The following question is only displayed to Type 1 Participants:

4.a. Think about your decision regarding allocations to the Transfer Account. Why did you decide to transfer as much as you did? Please indicate to what degree you agree with the following statements:

**I transferred as much as I did, because …**	**Strongly agree**	**Agree**	**Neutral**	**Disagree**	**Strongly disagree**
I followed the examples in the instructions. Type 1 participants that transferred less than other Type 1's received higher payoffs.					
I did not trust the Type 1 group members. I did not expect them to transfer much.					
I did not trust the Type 2 group members to allocate much to the Group Account.					
I did not expect allocations by the Type 1 members to the Transfer Account to increase the allocations by the Type 2 group members to the Group Account					
Because I cared about the other Type 1 members of my group first.					
To get the highest payoff for myself.					
To get the highest payoff for the whole group, both for Type 1 and Type 2.					
I wanted to transfer some but also save some.					
To support the Type 2 group members in their allocations to the Group Account.					
I felt the responsibility to make allocations to the Transfer Account so I would not let my group members down.					
Because allocating to the Transfer Account was the right thing to do, irrespective of what other Type 1 and Type 2 group members did.					
I did not understand the decision task of Type 1 participants.					

The following question is only displayed to Type 2 Participants:

4.b. Think about your decisions on contributions to the group account. Why did you decide to contribute as much as you did? Please indicate to what degree you agree with the following statements:

**I contributed as much as I did, because …**	**Strongly agree**	**Agree**	**Neutral**	**Disagree**	**Strongly disagree**
I followed the example in the instructions. Type 2 participants that contributed less than other Type 2's received higher payoffs.					
I thought that the aggregate transfers of Type 1 group members were high enough					
I did not trust much the Type 2 group members. I did not expect them to contribute much.					
To get the highest payoff for myself.					
Because I cared about my other Type 2 group members first.					
To get the highest payoff for the whole group, both for Type 1 and Type 2.					
I wanted to contribute some but also save some.					
I felt the responsibility to contribute so I would not let my group members down.					
Because contributing is the right thing to do, irrespective of what the Type 1 and Type 2 group members do.					
I did not understand the decision task of Type 2 participants.					

8. SI References

1. R. M. Isaac, J. M. Walker, S. H. Thomas, Divergent Evidence on Free Riding : An Experimental Examination of Possible Explanations. *Public Choice* **43**, 113–149 (1984).

2. J. O. Ledyard, Public Goods: A Survey of Experimental Research. *The Handbook of Experimental Economics, ed. by J. H. Kagel, A. Roth, Chapter 2, Princeton University Press.* 111–194 (1995). https://doi.org/10.1021/ed024p574.

3. J. Zelmer, Linear public goods experiments: A meta-analysis. *Exp Econ* **6**, 299–310 (2003).

4. A. Chaudhuri, Sustaining cooperation in laboratory public goods experiments: A selective survey of the literature. *Exp Econ* **14**, 47–83 (2011).

5. R. M. Isaac, J. M. Walker, A. W. Williams, Group size and the voluntary provision of public goods. Experimental evidence utilizing large groups. *J Public Econ* **54**, 1–36 (1994).

6. J. K. Goeree, C. A. Holt, S. K. Laury, Private costs and public benefits: Unraveling the effects of altruism and noisy behavior. *J Public Econ* **83**, 255–276 (2002).

7. J. Carpenter, S. Bowles, H. Gintis, S. H. Hwang, Strong reciprocity and team production: Theory and evidence. *J Econ Behav Organ* **71**, 221–232 (2009).

8. D. Nosenzo, S. Quercia, M. Sefton, Cooperation in small groups: the effect of group size. *Exp Econ* **18**, 4–14 (2015).

9. J. Weimann, J. Brosig-Koch, T. Heinrich, H. Hennig-Schmidt, C. Keser, Public good provision by large groups – the logic of collective action revisited. *Eur Econ Rev* **118**, 348–363 (2019).

10. T. Goeschl, S. E. Kettner, J. Lohse, C. Schwieren, How much can we learn about voluntary climate action from behavior in public goods games? *Ecological Economics* **171** (2020).

11. R. M. Isaac, J. M. Walker, Group size effects in public goods provision: The voluntary contributions mechanism. *Quarterly Journal of Economics* **103**, 179–199 (1988).

12. W. T. Harbaugh, K. Krause, Children's altruism in public good and dictator experiments. *Econ Inq* **38**, 95–109 (2000).

13. V. Lugovskyy, D. Puzzello, A. Sorensen, J. Walker, A. Williams, An experimental study of finitely and infinitely repeated linear public goods games. *Games Econ Behav* **102**, 286–302 (2017).

14. A. Gunnthorsdottir, D. Houser, K. McCabe, Disposition, history and contributions in public goods experiments. *J Econ Behav Organ* **62**, 304–315 (2007).

15. P. van den Berg, P. Dewitte, I. Aertgeerts, T. Wenseleers, How the incentive to contribute affects contributions in the one-shot public goods game. *Sci Rep* **10**, 8–12 (2020).

16. M. Bagnoli, M. Mckee, Voluntary Contribution Games: Efficient Private Provision of Public Goods. *Econ Inq* **29**, 351–366 (1991).

17. J. Fisher, R. M. Isaac, J. W. Schatzberg, J. M. Walker, "Heterogenous Demand for Public Goods: Behavior in the Voluntary Contributions Mechanism" *Public Choice*, **85**, 249-266 (1995).

18. K. S. Chan, S. Mestelman, R. Moir, R. A. Muller, "Heterogeneity and the Voluntary Provision of Public Goods" (1999). *Exp Econ* **2**, 5-30 (1999).

19. F. Tan, Punishment in a linear public good game with productivity heterogeneity. *Economist* **156**, 269–293 (2008).

20. E. Reuben, A. Riedl, Public goods provision and sanctioning in privileged groups. *Journal of Conflict Resolution* **53**, 72–93 (2009).

21. U. Fischbacher, S. Schudy, S. Teyssier, Heterogeneous reactions to heterogeneity in returns from public goods. *Soc Choice Welfare* **43**, 195–217 (2014).

22. J. Falkinger, E. Fehr, S. Gächter, R. Winter-Ebmer, "A Simple Mechanism for the Efficient Provision of Public Goods: Experimental Evidence" *American Economic Review* **91**, 247-264 (2000).

23. S. Jin, S. Columbus, P. A. M. van Lange, D. Balliet, Conflict, cooperation, and institutional choice. *J Exp Soc Psychol* **111** (2024).

24. E. Blanco, M. C. Lopez, J. M. Walker, The Opportunity Costs of Conservation with Deterministic and Probabilistic Degradation Externalities. *Environ Resour Econ (Dordr)* **64**, 255–273 (2016).

25. E. Blanco, T. Haller, M. C. Lopez, J. M. Walker, The tension between private benefits and degradation externalities from appropriation in the commons. *J Econ Behav Organ* **125**, 136–147 (2016).

26. B. Stoddard, Risk in payoff-equivalent appropriation and provision games. *Journal of Behavioral and Experimental Economics* **69**, 78–82 (2017).

27. S. Gächter, D. Marino Fages, "Using the Strategy Method and Beliefs to Explain Group Size and MPCR Effects in Public Good Experiments" [Preprint] (2023). https://durham-repository.worktribe.com/output/1748683

28. C. Gallier, *et al.*, Leveling up? An inter-neighborhood experiment on parochialism and the efficiency of multi-level public goods provision. *J Econ Behav Organ* **164**, 500–517 (2019).

29. M. Catola, S. D'Alessandro, P. Guarnieri, V. Pizziol, Multilevel public goods game: Levelling up, substitution and crowding-in effects. *J Econ Psychol* **97** (2023).

30. G. Charness, L. Rigotti, A. Rustichini, Social surplus determines cooperation rates in the one-shot Prisoner's Dilemma. *Games Econ Behav* **100**, 113–124 (2016).

31. V. Capraro, J. J. Jordan, D. G. Rand, Heuristics guide the implementation of social preferences in one-shot Prisoner's Dilemma experiments. *Sci Rep* **4** (2014).

32. N. Gupta, L. Rigotti, A. Wilson, The Experimenters' Dilemma: Inferential Preferences over populations. *arXiv* [Preprint] *https://doi.org/10.48550/arXiv.2107.05064.* (2021).

33. S. Gächter, K. Lee, M. Sefton, T. O. Weber, The role of payoff parameters for cooperation in the one-shot Prisoner's Dilemma. *Eur Econ Rev* **166** (2024).

34. R. M. Isaac, D. Schmidtz, J. M. Walker, The Assurance Problem in a Laboratory Market. *Public Choice* **62**, 217–236 (1989).

35. R. Suleiman, A. Rapoport, Provision of step-level public goods with continuous contribution. *J Behav Decis Mak* **5**, 133–153 (1992).

36. C. B. Cadsby, E. Maynes, Voluntary provision of threshold public goods with continuous contributions: Experimental evidence. *J Public Econ* **71**, 53–73 (1999).

37. R. Croson, M. Marks, Identifiability of Individual Contributions in a Threshold Public Goods Experiment. *J Math Psychol* **42**, 167–190 (1998).

38. M. B. Marks, R. T. A. Croson, The effect of incomplete information in a threshold public goods experiment. *Public Choice* **99**, 103–118 (1999).

39. R. T. A. Croson, M. B. Marks, Step returns in threshold public goods: A meta- and experimental analysis. *Exp Econ* **2**, 239–259 (2000).

40. D. L. Dickinson, The voluntary contributions mechanism with uncertain group payoffs. *J Econ Behav Organ* **35**, 517–533 (1998).

41. L. Gangadharan, V. Nemes, Experimental analysis of risk and uncertainty in provisioning private and public goods. *Econ Inq* **47**, 146–164 (2009).

42. E. Blanco, T. Haller, J. M. Walker, Externalities in appropriation: responses to probabilistic losses. *Exp Econ* **20**, 793–808 (2017).

43. C. Noussair, C. Soo, Voluntary contributions to a dynamic public good: Experimental evidence. *Econ Lett* **98**, 71–77 (2008).

44. I. M. Buso, *et al.*, Lab-like findings from online experiments. *J Econ Sci Assoc* **7**, 184–193 (2021).

45. J. Hergueux, N. Jacquemet, Social preferences in the online laboratory: a randomized experiment. *Exp Econ* **18**, 251–283 (2015).

46. M. Marks, R. Croson, Alternative rebate rules in the provision of a threshold public good: An experimental investigation. *J Public Econ* **67**, 195–220 (1998).

47. M. A. Spencer, S. K. Swallow, J. F. Shogren, J. A. List, Rebate rules in threshold public good provision. *J Public Econ* **93**, 798–806 (2009).

48. D. Rondeau, W. D. Schulze, G. L. Poe, Voluntary revelation of the demand for public goods using a provision point mechanism. *J Public Econ* **72**, 455–470 (1999).

49. E. Blanco, N. Struwe, J. M. Walker, Experimental evidence on sharing rules and additionality in transfer payments. *J Econ Behav Organ* **188**, 1221–1247 (2021).

50. R. T. A. Croson, "Thinking like a game theorist: factors affecting the frequency of equilibrium play". *J Econ Behav Organ* **41**, 299-314 (2000).

51. R. T. A. Croson, Theories of commitment, altruism and reciprocity: Evidence from linear public goods games. *Econ Inq* **45**, 199–216 (2007).

52. O. Bock, I. Baetge, A. Nicklisch, Hroot: Hamburg Registration and Organization Online Tool. *Eur Econ Rev* **71**, 117–120 (2014).

53. U. Knief, W. Forstmeier, Violating the normality assumption may be the lesser of two evils. *Behav Res Methods* **53**, 2579–2590 (2021).

54. T. Lumley, P. Diehr, S. Emerson, L. Chen, The importance of the normality assumption in large public health data sets. *Annu. Rev. Public Health* **23**, 151–69 (2002).

55. J. A. List, A. M. Shaikh, Y. Xu, Multiple hypothesis testing in experimental economics. *Exp Econ* **22**, 773–793 (2019).
